# Generating simple classification rules to predict local surges in COVID-19 hospitalizations

**DOI:** 10.1007/s10729-023-09629-4

**Published:** 2023-01-24

**Authors:** Reza Yaesoubi, Shiying You, Qin Xi, Nicolas A. Menzies, Ashleigh Tuite, Yonatan H. Grad, Joshua A. Salomon

**Affiliations:** 1 Department of Health Policy and Management, Yale School of Public Health, 350 George Street, Room 308, New Haven, CT 06510 USA; 2 Public Health Modeling Unit, Yale School of Public Health, New Haven, CT USA; 3 Department of Global Health, Harvard T.H. Chan School of Public Health, Boston, MA USA; 4 Epidemiology Division, University of Toronto Dalla Lana School of Public Health, Toronto, Ontario Canada; 5 Department of Immunology and Infectious Diseases, Harvard T. H. Chan School of Public Health, Boston, MA USA; 6 Division of Infectious Diseases, Brigham and Women’s Hospital, Harvard Medical School, Boston, MA USA; 7 Department of Health Policy, Stanford University School of Medicine, Palo Alto, CA USA

**Keywords:** Surveillance, Prediction, Decision tree, Machine learning, Simulation, COVID-19

## Abstract

Low rates of vaccination, emergence of novel variants of SARS-CoV-2, and increasing transmission relating to seasonal changes and relaxation of mitigation measures leave many US communities at risk for surges of COVID-19 that might strain hospital capacity, as in previous waves. The trajectories of COVID-19 hospitalizations differ across communities depending on their age distributions, vaccination coverage, cumulative incidence, and adoption of risk mitigating behaviors. Yet, existing predictive models of COVID-19 hospitalizations are almost exclusively focused on national- and state-level predictions. This leaves local policymakers in urgent need of tools that can provide early warnings about the possibility that COVID-19 hospitalizations may rise to levels that exceed local capacity. In this work, we develop a framework to generate simple classification rules to predict whether COVID-19 hospitalization will exceed the local hospitalization capacity within a 4- or 8-week period if no additional mitigating strategies are implemented during this time. This framework uses a simulation model of SARS-CoV-2 transmission and COVID-19 hospitalizations in the US to train classification decision trees that are robust to changes in the data-generating process and future uncertainties. These generated classification rules use real-time data related to hospital occupancy and new hospitalizations associated with COVID-19, and when available, genomic surveillance of SARS-CoV-2. We show that these classification rules present reasonable accuracy, sensitivity, and specificity (all ≥ 80%) in predicting local surges in hospitalizations under numerous simulated scenarios, which capture substantial uncertainties over the future trajectories of COVID-19. Our proposed classification rules are simple, visual, and straightforward to use in practice by local decision makers without the need to perform numerical computations.

**Supplementary Information:**

The online version contains supplementary material available at 10.1007/s10729-023-09629-4.

## Highlights

- Low rates of vaccination, emergence of novel variants of SARS-CoV-2 (such as the omicron variant), and increasing transmission relating to seasonal changes leave many U.S. communities at risk for surges of COVID-19
- Centers for Disease Control and Prevention (CDC) created the COVID-19 Community Levels framework to identify the potential for strain on the local health systems. The risk classification rules proposed by this framework, however, are not explicitly linked to the outcome of interest, which is whether the local healthcare capacity is expected to be surpassed due to COVID-19 hospitalizations.
- To address this need, our study describes a method to identify simple and easy-to-communicate classification rules that can provide early warnings when a pre-specified threshold of hospital capacity is likely to be exceeded within a 4- or 8-week period.
- These simple classification rules can be used by local decision makers to utilize data from existing surveillance systems to proactively respond to surges in COVID-19 hospitalizations.

## Introduction

Many communities are at risk of surging COVID-19 hospitalizations due to low rates of vaccination, emergence of novel variants of SARS-CoV-2, and seasonal changes in transmission [1]. Understanding the likely trajectory of the pandemic and its implications for demands on the healthcare system are important for policymakers aiming to prepare for and possibly prevent surges that result in hospital demand that exceeds capacity [2]. Since the beginning of the pandemic, substantial efforts have been invested in developing models to predict the trajectories of cases, hospitalizations, and deaths associated with COVID-19 (e.g., COVID-19 Forecast Hub [3] or the IHME COVID-19 Forecasting Model [4]). While the spread of SARS-CoV-2 and hospitalizations due to COVID-19 vary substantially across different geographic regions (as influenced by a population’s characteristics, local policies, and adoption of risk-mitigating behaviors), these models typically focus on predictions at national or state levels. This leaves local policymakers in urgent need of tools that can signal when risks are high for overwhelming local hospital capacity with COVID-19 cases in the absence of additional mitigation measures.

To address this need, the Centers for Disease Control and Prevention (CDC) created the COVID-19 Community Levels framework to identify the potential for strain on the local health systems [5]. The risk classification rules proposed by this framework, however, are not explicitly linked to the outcome of interest, which is whether the local healthcare capacity is expected to be surpassed due to COVID-19 hospitalizations.

Local trajectories of COVID-19 hospitalizations are impacted by various factors, including the proportion of the population with infection- or vaccine-induced immunity, the duration of infection- and vaccine-induced immunity, uptake and effectiveness of vaccine boosters, the transmissibility, immune evasion, and virulence of novel variants (such as the omicron variant and subvariants) that may continue to emerge and spread, the effectiveness of vaccines against prevalent strains including novel variants, and population behavior and adherence to mitigating strategies [1, 2, 6–9]. True values of these pandemic parameters and state variables are either unobservable or can only be estimated with a high level of uncertainty, which further challenges our ability to predict local trajectories of COVID-19 pandemic [2].

Data from hospital occupancy censuses, rate of new COVID-19 hospital admissions, and vaccination coverage are often available to monitor the local spread of SARS-CoV-2 and trends in COVID-19 hospitalizations [5]. To enable local policymakers to translate the data from these surveillance systems into timely decisions, this study aimed to identify simple and easy-to-communicate classification rules to provide early warnings when a pre-specified threshold of hospital capacity is likely to be exceeded within a 4- or 8-week period. To identify these classification rules, we developed a model of SARS-CoV-2 transmission that incorporates complexities, changes, and uncertainties regarding the biology of SARS-CoV-2 and factors driving local trajectories of COVID-19 in the past and future. We used this model to generate simulated trajectories of COVID-19 hospitalizations to train classification decision tress, which present interpretable classification rules to predict local surges in COVID-19 hospitalizations. We further evaluated the robustness of these classification rules’ accuracy using simulated scenarios, which capture substantial uncertainties over the future trajectories of COVID-19 at the local level. classification rule.

## Methods

### Overview

Multiple indicators are collected through surveillance systems to monitor and predict local trends in COVID-19 hospitalizations (Table 1). We use these indicators (which we will refer to as ‘features’) to summarize the information from each surveillance system into a number of predictors (e.g., the average change in the number of hospitalizations during the past 4 weeks or the number of individuals vaccinated thus far). For seasonal infectious diseases (e.g., seasonal influenza), a main goal of developing predictive models is to predict demand curves (e.g., demand for hospital beds or for antiviral drugs over a certain period). For a novel pathogen or one that has not yet settled into predictable endemic cycles (such as SARS-CoV-2), which could potentially overwhelm the health care system, an equally important goal is to develop an alert system to predict whether such event could occur in short term. The significance of these alert systems is to assist policymakers to decide whether to trigger non-pharmaceutical measures such as limiting mass gathering events or closing schools/workspaces to curb the spread of the novel pathogen. As such, our goal in this study was to develop decision trees to predict if the local hospitalization capacity will be surpassed within 4 or 8 weeks based on the values of features defined in Table 1. Decision tree models provide simple, visual, and explicit classification rules to predict the outcomes of interest, which makes them straightforward to use in practice [10, 11].

**Table 1:** Observations available through surveillance systems to predict the local trend in COVID-19 hospitalizations

Surveillance	Features used for prediction
Rate of hospital occupancy due to COVID-19	- Current value
Weekly rate of new hospital admissions due to COVID-19	- Average over the past 2 weeks - Average change during the past 4 weeks
Vaccination coverage	- Cumulative value
Prevalence of novel variant among new infections	- Average over the past 2 weeks - Average change during the past 4 weeks

Predictive models are usually trained on historical data. If the process that generates data does not substantially change over time, models trained on historical data could provide accurate predictions in the future. In the context of COVID-19 pandemic, however, the assumption of a stationary data-generating process does not necessarily hold. The factors impacting the observations related to COVID-19 (e.g., the timing and the effectiveness of mitigating strategies, the characteristics of novel variants, and the coverage of vaccination among different age groups) will most likely continue to change in the future. The types and the effectiveness of mitigating strategies during the near future could be markedly different than those employed in the past, novel variants such as omicron may gain hold over different time courses in different locations, and their characteristics in terms of transmissibility and virulence will be highly uncertain during their initial seeding, and vaccination coverage trends are also uncertain and contingent. Hence, to develop decision trees that are robust against changes in the data generating process and future uncertainties, we used epidemic trajectories simulated by a model of SARS-CoV-2 transmission in the U.S. between March 1, 2020 and June 1, 2022 to train and evaluate our decision trees.

**Fig. 1:**
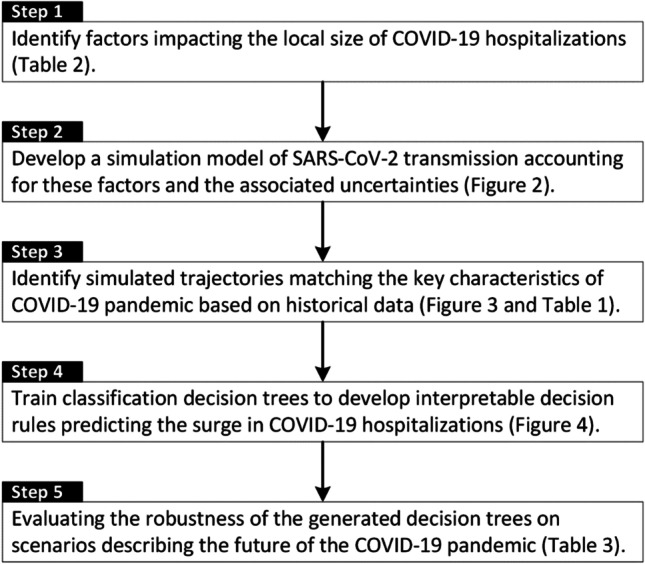
A framework to generate simple, interpretable classification rules to predict local surges in COVID-19 hospitalizations

This simulation model is structured to incorporate factors, and the associated uncertainties, that impact the local size of COVID-19 hospitalizations during the winter 2021–2022 and spring of 2022 (Table 2). To build the datasets needed for our purpose, we used a set of simulated trajectories that satisfy specific epidemiological conditions during March 1, 2020 and November 30, 2021. These conditions, which relate to the historical rate of hospitalization (overall and by age), age-distribution of hospitalizations, the prevalence of population with immunity against SARS-CoV-2, the spread of the delta variant, and the rate of vaccination (overall and by age), ensured that the selected trajectories are consistent with past trajectories of COVID-19. We then projected these selected trajectories onto the period of the winter 2021–2022 and spring of 2022 to build the datasets needed to train and evaluate our decision trees. Our proposed framework to identify and evaluate these classification rules is depicted in Fig. 1 and the details of this simulation model and the process to select trajectories are provided below.

**Table 2:** Factors that could influence the local trajectory of COVID-19 hospitalizations [1, 2, 6–9]

Epidemic parameters
Size and age-distribution of the population
${R}_{0}$ of the dominant and novel strains
Seasonality
Transmissibility of the dominant strain and the novel variant
Virulence of the dominant strain and the novel variant (may vary by age)
Duration of infectiousness for the dominant strain and the novel variant
Duration of hospitalization for the dominant strain and the novel variant
Duration of infection-induced immunity for the dominant strain and the novel variant
Duration of vaccine-induced immunity for the dominant strain and the novel variant
Effectiveness of vaccine against infection and hospitalizations
Effectiveness of vaccine in reducing infectiousness for the dominant strain and the novel variant
Duration and effectiveness of non-pharmaceutical interventions ever used
Overlap between infection-induced immunity and vaccine-induced immunity (i.e., does vaccination increase the duration of immunity from infection?)
Extent to which previous infections from one variant offers immunity against others
Epidemic state variables
Proportion of population vaccinated
Proportion of population with infection- or vaccine-induced immunity

### A simulation model of SARS-CoV-2 transmission

**Fig. 2:**
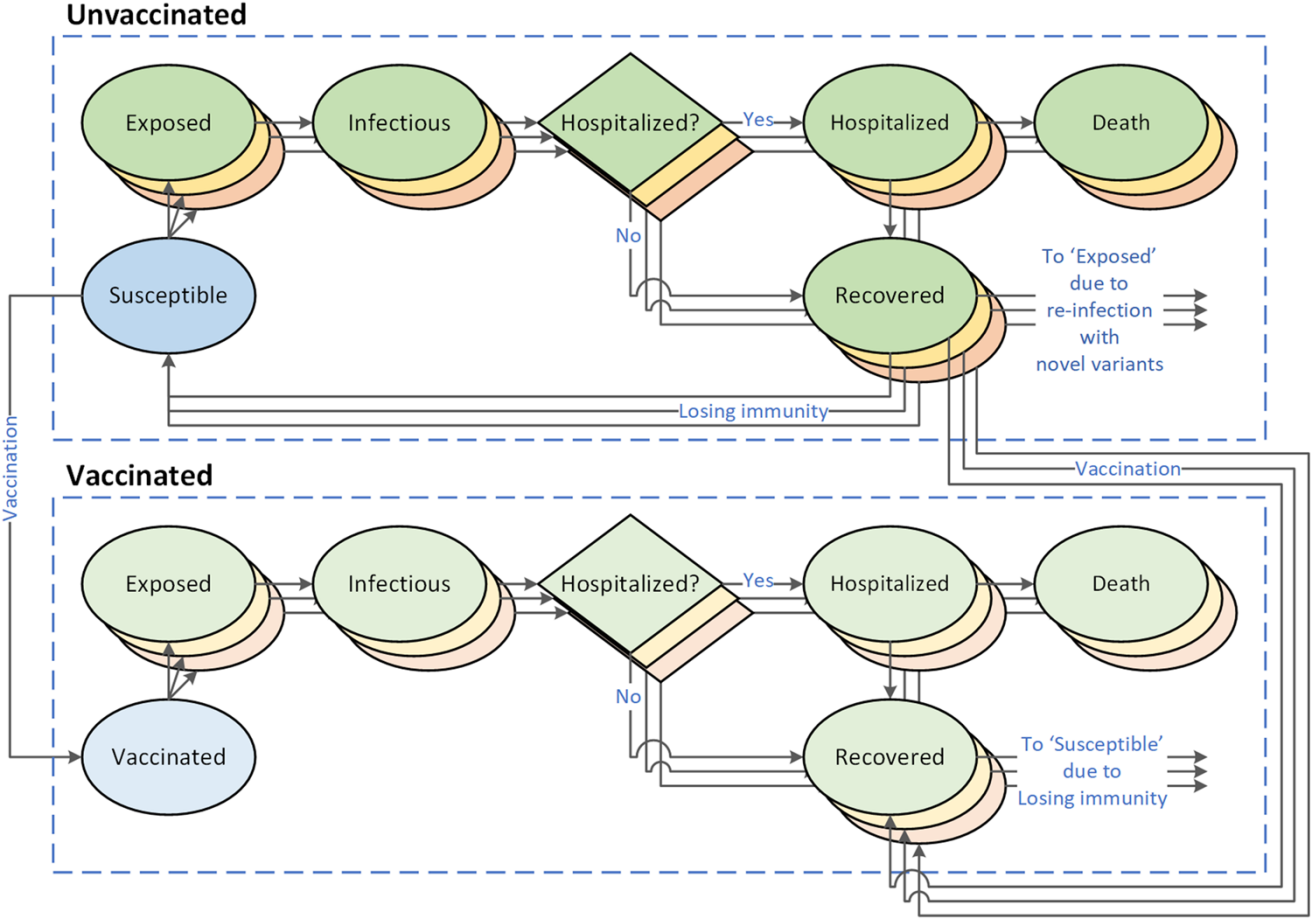
A stochastic, age-structured model of SARS-COV-2 transmission with three strains and two vaccination status. The green, yellow, and red compartments represent, respectively, the ancestral strain of SARS-CoV-2, the delta variant, and a novel variant of SARS-CoV-2 (such as the omicron variant) that might emerge and spread

We developed a stochastic, age-structured model that describes the transmission of three main variants of SARS-CoV-2 between March 1, 2020 and June 1, 2022 (Fig. 2). The variants represent the ancestral strain of SARS-CoV-2 that dominated during 2020, the delta variant that began spreading in the Spring of 2021, and a novel variant, such as omicron, that overtook delta [12, 13]. The model projects the weekly incidence of cases, hospitalization, and deaths due to COVID-19 among age groups 0–4, 5–12, 13–17, 18–29, 30–49, 50–64, 65–74, and 75 + in communities with population between 250,000 and 1,250,000. The mixing patterns between age groups are modeled using the age-specific contact rates estimated for the U.S. population [14] (see §S2.3 of the Supplement).

As the model attempts to describe the transmission of SARS-CoV-2 at the local level, we allowed for a continuous importation of cases from neighboring communities. An imported case could be infected with the novel variant with a probability that begins to increase around December 2021 according to a sigmoid function (Fig. S1). Given the uncertainty in the timing for the introduction of the novel variant, we allowed the magnitude of this probability and the rate at which it increases over time to vary across simulated trajectories (Fig. S1 and Table S3).

We assumed that compared to the current dominant strain, the novel variant could be more transmissible (up to twice) [15, 16], could lead to milder or more severe disease (up to 200% increased or 100% decreased probability of hospitalization) [13, 17, 18], could cause a shorter or longer duration of infectiousness (up to 100% increase or decrease), and could evade immunity conferred by previous infection or vaccination (Table S3 and Table S4).

We assumed that vaccination began in December 2020 at an age-specific rate that gradually decreased over time (Fig. S2 and Table S5). For the ancestral strain of SARS-CoV-2, vaccine provided 85%-100% effectiveness against hospitalization and reduced the duration of infectiousness by 25%-75% [19–21]. Our model does not differentiate vaccinated individuals based on the type of vaccine or the number of vaccine doses they have received; therefore, we consider an individual “vaccinated” when they can be assumed to have reached the level of immunity described above. We assumed that vaccine-induced immunity wanes (within 0.5–2.5 year) leading to the vaccinated individual becoming susceptible (Fig. 2).

With respect to the novel variant, vaccinated individuals were assumed to have partial immunity to infection (up to 100%), and if infected, experience a shorter duration of infectiousness by 25%-50% [21] and are 50–100% less likely to require hospitalization (Table S5). We also assumed that vaccination increases the duration of infection-induced immunity by up to 50% for both the dominant strain and novel variant (Table S5).

To model the effect of control measures and population adherence to public health recommendations across different communities and since the beginning of the pandemic, we assumed that control measures went into effect whenever the rate of hospital occupancy due to COVID-19 exceeded the threshold ${T}_{1}$ and were lifted whenever this rate dropped below the second threshold ${T}_{2}$ [22]. We further assumed that the intensity and the effectiveness of control measures in reducing the effective reproductive number increase with the rate of hospital occupancy according to some sigmoid function (Fig. S3). To account for the variation in timing and effectiveness of control measure across different communities, we allowed the thresholds ${T}_{1}$ and ${T}_{2}$, and the function that models the effectiveness of control measure to vary across simulated trajectories and to be determined by random draws from appropriate probability distributions (see §S2.4 of Supplement).

### Modeling errors in surveillance estimates

The estimates provided by certain surveillance systems are subject to error due to limited or unrepresentative samples. Among the surveillance systems of Table 1, we assumed that the rate of hospital occupancy, the weekly rate of new hospitalizations, and vaccination coverage can be observed with no error in each community. The accuracy of estimates for the prevalence of a novel variant among the new infections depends on the number of samples collected and tested. To account for this sampling error, we used the following approach. Let ${y}_{t}$ be the true value of the prevalence of a novel variant among infections in week $t$ of the pandemic. We assume that ${y}_{t}$ can be observed (denotated by ${\widehat{y}}_{t})$ with some error (${\epsilon }_{t})$ and a delay of one week [23]:

$${\widehat{y}}_{t}={y}_{t-1}+{\epsilon }_{t}.$$

Here, we assume that ${\epsilon }_{t}$ follows a normal distribution with mean 0 and standard deviation $\sqrt{{y}_{t}(1-{y}_{t})/N}$, where $N$ is the sample size of the survey. A higher value of $N$ decreases the variance of the error ${\epsilon }_{t}$ leading to more accurate estimates. The exact number of samples that are tested for novel variants per week is unclear [23]; therefore, we assumed that enough samples are collected to estimate the prevalence of 1% with the 95% confidence interval of (0.5–1.5%). This requires a sample size of $N=1521$ per week.

### Selection of simulated trajectories to develop and evaluate decision tree models

To ensure that the trajectories simulated by our model were consistent with the community-level spread of SARS-CoV-2 in the U.S., we used a likelihood approach to measure the fit of each simulated trajectory against the data related to the following outcomes:

1. **Prevalence of population with immunity from infection**: The CDC’s seroprevalecnce survey estiamtes that on average 20.6% of the U.S. population had immunity from infection on Auguest 26, 2021 with the state-level mimimum of 1.6% and the maximum of 34.1%. To measure how well a simulated trajectory matches these estimates, we estimate the likelihood of observing the seroprevalence of $\widehat{\mu }=20.6\%$, if the simulation trajectory results in the seroprevalence of $\mu$ using:
  $$L_1=f\left(x=\mu;\;\widehat\mu,\widehat\sigma\right),$$
  where $f$ is the probability density function of a normal distribution with mean $\widehat{\mu }$ and standard deviation of $\widehat{\sigma }=(34.1\mathrm{\%}-1.6\mathrm{\%})/4$. We only considered trajectories where the prevalence of population with immunity from infection does not surpass 35%, as informed by the CDC’s seroprevalecnce survey [24].
2. **Cumulative hospitalization rate**: To measure how well a simulated trajectory matches the observed data on cumulative hospitalization rate (i.e., the overall cumulative hospitalization rate of 768.0 per 100,000 population, with minimum of 301.7 and maximum of 1050.3 observed in the states included in COVID-NET, Table S7), we calculate the livelihood of this observation assuming that the simulated trajectory represents the reality. To this end, we measure the likelihood of observing the cumulative hospitalization rate of $\widehat{\mu }=$ 768.0 per 100,000 population, if the simulation trajectory results in the cumulative hospitalization rate of $\mu$ using:
  $$L_2=f\left(x=\mu;\;\widehat\mu,\widehat\sigma\right),$$
  where $f$ is the probability density function of a normal distribution with mean $\widehat{\mu }$ and standard deviation of $\widehat{\sigma }=(1050.3 -301.7)/4$.
3. **Cumulative hospitalization rate by age**: We used the same approach as described above to calculate the likelihood of observing hospitalization rates in each age group, as reported in Table S7. This returns likelihoods ${L}_{\mathrm{3,1}}, {L}_{\mathrm{3,2}},\dots , {L}_{\mathrm{3,8}}$ for 8 age groups included in our model.
4. **Cumulative vaccination rate**: We used the same approach as described above to calculate the likelihood $({L}_{4})$ of observation related to vaccination rates as reported in Table S9.
5. **Prevalence of the delta variant among new infections**: We used the same approach describe above to calculate the likelihood (${L}_{5}$) of observations related to the prevalence of the delta variant among new infections (Table S10).
6. **Weekly rate of hospital occupancy associated with COVID-19**: We only considered trajectories where the weekly hospital occupancy rate reaches at least 1.1 per 100,000 population but does not surpass 61.1 per 100,000 population. These thresholds are informed by hospital occupancy associated with COVID-19 in U.S. states during the period April 1 and July 7, 2020 [25].
7. **Weekly rates of new hospitalizations**: We only considered trajectories where the weekly rate of new hospitalizations reaches at least ${T}_{1}$ but does not surpass ${T}_{2}$ per 100,000 population, where ${T}_{1}$ is 0.75 times the minimum rate of new hospitalizations and ${T}_{2}$ is 1.25 times the maximum rate of new hospitalizations observed in the surveillance sites of COVID-Net (Table S7).

We calculated the natural logarithm of the likelihood of a trajectory as:

$$\mathrm{ln}\;\mathcal{L}=\mathrm{ln}\;{L}_{1}+\mathrm{ln}\;{L}_{2}+{\sum }_{k=1}^{8}\mathrm{ln}\;{L}_{3,k}+\mathrm{ln}\;{L}_{4}+\mathrm{ln}\;{L}_{5}.$$

We note that our goal is not to identify trajectories that exactly replicate the historical data related to hospitalizations, but instead, to consider simulated trajectories that meets the feasibility bounds described above. Therefore, to ensure that the overall burden of hospitalization was consistent with the data, we incorporated the likelihood ${L}_{2}$ when measuring the fit of a trajectory. To build a set of trajectories to train predictive models, we simulated as many trajectories as needed to obtain 7,500 feasible trajectories. For each simulated trajectory, parameter values were randomly drawn from the probability distribution of epidemic parameters listed in Table S2-Table S5. These prior distributions were informed by estimates extracted from existing scientific literature when such estimates are available; when not available, we assumed biologically-feasible distributions. Among the total of 293,193 simulated trajectory, we discarded 285,693 trajectories that violated the feasibility conditions described above and calculated the above pseudo-likelihood function for the remaining trajectories. After calculating $\mathrm{ln}\mathcal{L}$ for each simulation trajectory, we used 2,000 trajectories randomly selected among trajectories with a positive $\mathrm{ln}\mathcal{L}$ to train the predictive models.

This selection approach aimed to identify simulated trajectories that were consistent with the actual state-level trajectories of COVID-19 in the U.S. but also accommodated the additional variation and uncertainties in the trajectories of COVID-19 across more granular geographic regions. Here, each selected trajectory represents a community with unique values for the population characteristics (e.g., size and age distribution), effect of mitigating strategies, and other factors that determine the trajectory of COVID-19 hospitalizations during the winter 2021–2022 and spring of 2022 (as described in Table 2).

### Decision tree models

We considered two decision tree models which differed based on the surveillance systems they use to predict whether the hospital occupancy due to COVID-19 would surpass the hospitalization capacity in the next 4 or 8 weeks over the winter and spring months. Decision Tree A uses the information related to the current hospital occupancy, the weekly rate of new hospitalizations, and the vaccination coverage at the time of prediction. Decision Tree B augments Decision Tree A by assuming access to the percentage of weekly incidence due to novel variant, available through genomic surveillance of SARS-Co-V-2 [26].

We trained each model to predict whether hospital occupancy due to COVID-19 will exceed the hospitalization capacity of 15 per 100,000 population [27] within the next 4 or 8 weeks. We also established classification rules when hospitalization capacity is 10 or 20 per 100,000 population. To avoid overfitting, we used a minimal cost-complexity pruning approach [11], where we determined the complexity parameter using tenfold cross-validation to maximize the model accuracy (defined as the fraction of correct predictions) [10]. We used 2,000 simulated trajectories to train and optimize the parameters of each decision tree and used a separate set of 500 simulated trajectories to evaluate the final accuracy of each model. To build the datasets to develop and validate our predictive models, for each simulated trajectory, we recorded the values of features defined in Table 1 at weeks 0, 2, 4, …, 16, and 20 after the start of winter 2021–2022. For each recording, the outcome of interest to predict was whether the hospital occupancy would surpass a prespecified threshold within 4 or 8 weeks.

In addition to the estimated accuracy of each decision tree model, we also report the model’s sensitivity (i.e., the probability of correctly predicting the event where hospitalization capacity will be surpassed within the projection period of 4 or 8 weeks), and specificity (i.e., the probability of correctly predicting the event where hospitalization capacity will not be surpassed within the projection periods). For each model, we estimated the accuracy, sensitivity, and specificity using a separate set of simulated trajectories not used to train these models.

### Sensitivity analyses

While we developed and validated our decision trees using a wide range of simulated trajectories, we also evaluated whether the accuracy of our predictive models persists under three extreme scenarios:

1. In our main analysis, we assumed that some forms of non-pharmaceutical measures (e.g., physical distancing and mask use recommendations) with varying degrees of effectiveness would remain in effect during winter 2021–2022 and spring of 2022. Our first sensitivity analysis considered a scenario in which all non-pharmaceutical measures are removed (or the adherence to public health recommendations is minimal due to public fatigue) during this period [1].
2. In our main analysis, we assumed that a novel variant with uncertain degree of transmissibility and virulence emerges and spreads during winter 2021–2022 and spring of 2022. Our second sensitivity analysis considers the scenario where no novel variant spreads during this period.
3. Finally, we trained our predictive models assuming that the prevalence of novel variant among new infections are estimated using the sample size of $N=1521$ test per week (as described above, this was calculated based on the assumption that enough samples are collected to estimate the 1% prevalence of the novel variant with the 95% confidence interval of (0.5–1.5%)). Our third sensitivity analysis considered the scenario where a smaller number of samples ($N=250$) are tested for infection with novel variant.

## Results

**Fig. 3:**
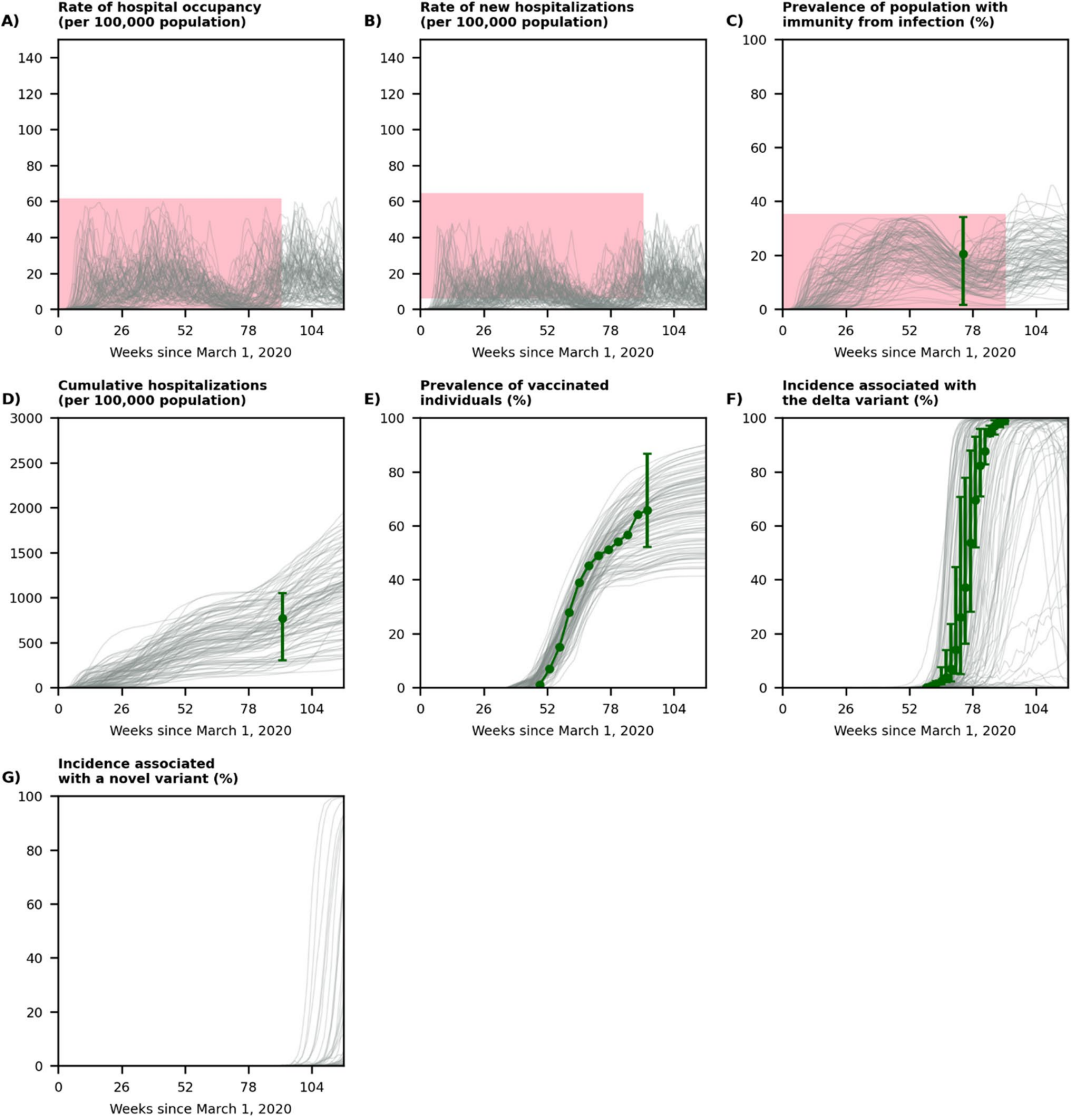
Displaying a random set of 100 trajectories simulated by our model (out of 1,000 simulated trajectories used to develop our decision trees). The week 91 marks the beginning of winter 2022. The red regions represent the feasibility conditions for including a simulated trajectory to train and evaluate the predictive models (see Methods for details). The green dot in **panel C** is the prevalence of individuals with immunity againts SARS-CoV-2 and the interval represent the minimum and maximum prevalence in U.S. states as estimated by the CDC’s seroprevalence survey [24]. The green dot in panel **D** is the cumulative hospitalization rate in the U.S. and the interval represents the minimum and maximum cumulative hospitalization rates observed in the surveillance sites of COVID-NET on November 27, 2021 (Table S7). The green dots in panel **E** represent the vaccination coverage provided by COVID data tracker, defined as the percentage of the population fully vaccinated (Table S8) and the interval represented the minimum and maximum vaccination coverage in all states (Table S9) on December 7, 2021. The green dots in panel **F** represent the prevalence of delta variant among new cases estimated by the CDC’s COVID Data Tracker; the intervals represent the minimum and the maximum values observed among 10 U.S. regions (Table S10). See Fig. S5 for the behavior of selected trajectories with respect to age-specific targets

Figure 3 demonstrates that our selected simulated trajectories to train our decision trees were consistent 1) with historical data on hospitalizations due to COVID-19, vaccination coverage in the U.S., and the spread of the delta variant; and 2) with the latest data regarding the state of the pandemic at the beginning of winter 2021–2022 (i.e., week 91 in Fig. 3). The red regions in Fig. 3A-C represent the feasibility conditions for including a simulated trajectory to train and evaluate our predictive models with respect to the weekly rates of hospital occupancy, new hospitalizations, and the prevalence of population with immunity from infection. The age-specific rates of cumulative hospitalization and vaccination as well as the age-distribution of cumulative hospitalizations in our selected trajectories were also consistent with the reported data (Fig. S5), which confirms the ability of our model to capture the transmission of SARS-CoV-2 among different U.S. age groups. Figure 3C-E show that our selected trajectories were representative of the state of the pandemic at the end of November 2021 as determined by the prevalence of population with immunity from infection (Fig. 3C), the rate of cumulative hospitalization (Fig. 3D), the prevalence of vaccinated individuals (Fig. 3E).

With respect to the spread of novel variants, Fig. 3F compares the proportion of weekly incidence associated with the delta variant in our selected trajectories with the estimated prevalence of the delta variant in the U.S. during April and August 2021. Figure 3G displays the potential spread of a novel variant starting in the winter. For some trajectories, the spread of the novel variant was similar to that of the delta variant in the U.S., but for others, the spread was faster or slower depending on the characteristics of the novel variant.

There were substantial variations across our selected trajectories induced by various uncertain factors that influence the medium-term future trajectory of COVID-19 (Table 2). Among our selected trajectories, 92.3%, 82.1%, and 70.2% surpassed the hospitalization capacities of 10, 15, and 20 per 100,000 population during the winter and spring (Fig. 3A); the peak of hospital occupancy due to COVID-19 was in 95^th^ percentile range (4.8, 59.2) with mean 29.4 per 100,000 population (Fig. 3A); the peak of new hospital admission rate was in 95^th^ percentile range (4.2, 51.7) with mean 24.8 (Fig. 3B); and the prevalence of the population with immunity from infection varied between (0.8%, 50.8%) (Fig. 3C). Among these trajectories, by July 1, 2022 the rate of cumulative hospitalization since the beginning of the pandemic would be in 95^th^ percentile range (267.2, 1800.9) with mean 983.6 per 100,000 population (Fig. 3D), and the prevalence of vaccinated individuals would be in the 95^th^ percentile range (44.8, 90.5) with mean 68.7 (Fig. 3E). The prevalence of novel variant among new infections reached 5% among 15.6% of selected trajectories and reached 95% among 3.5% of the selected trajectories (Fig. 3F) during the winter 2021–2022 and spring of 2022.

The final dataset we used to develop our decision tree models included 7000 records and the hospital capacity surpassed the thresholds of 10, 15, and 20 per 100,000 population within 8 weeks in 82.1%, 66.8%, and 51.4% of these simulations. The correlations between the features defined in Table 1 and the event that hospital capacity surpassed the above thresholds within 4 or 8 weeks were all significant (Table S11-Table S12).

**Fig. 4:**
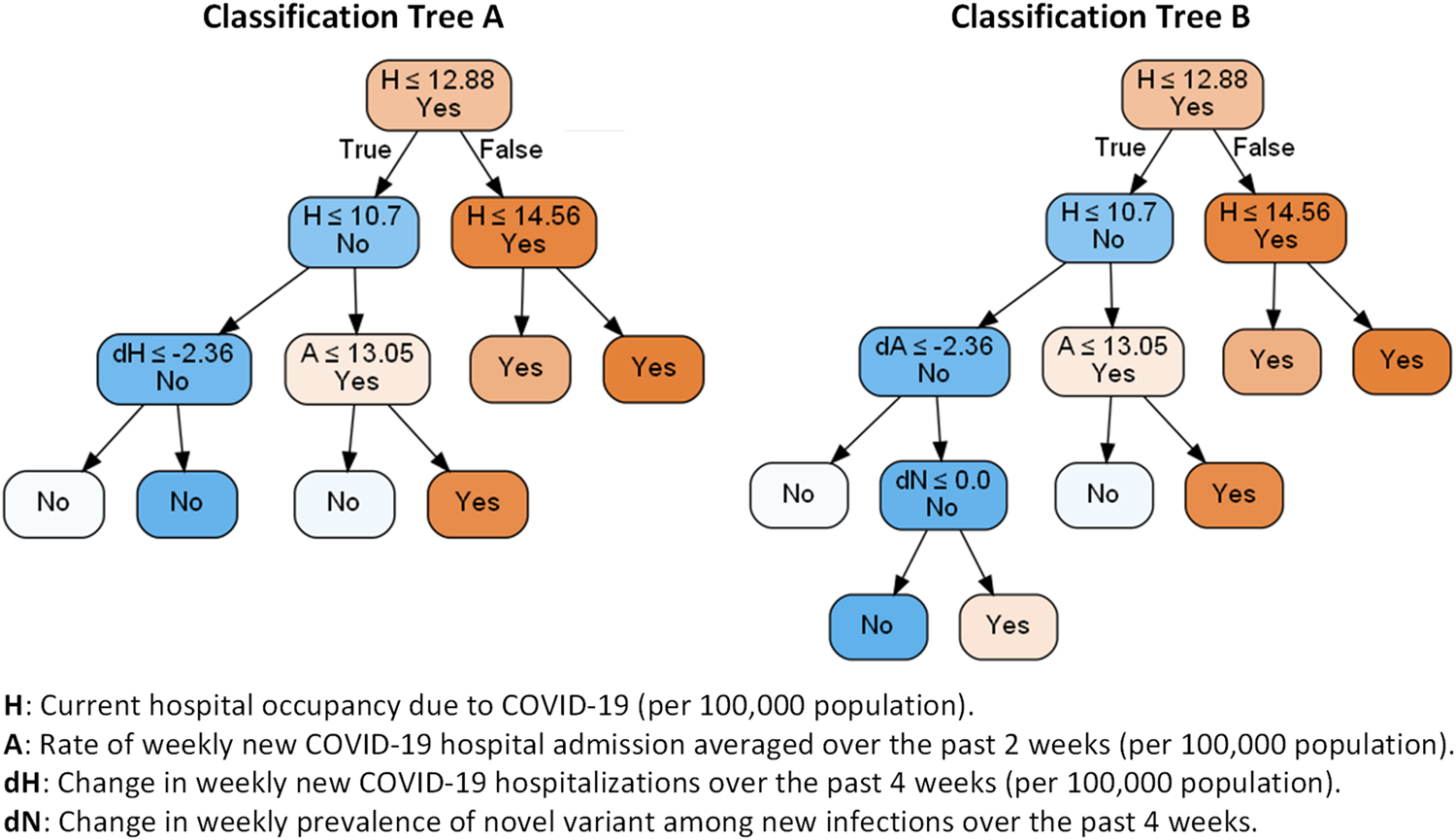
Decision Trees A and B to predict whether the hospital occupancy due to COVID-19 would surpass the threshold of 15 per 100,000 population within the next 8 weeks during the winter and spring of 2022. ‘Yes’ denotes the prediction that hospital occupancy will surpass the capacity and ‘No’ denotes the prediction that hospital occupancy will remain below the capacity. Between two descendent nodes, darker color indicates higher proportion of observations reaching the node. Decision trees for hospitalization capacity of 10 and 20 per 100,000 population are shown in Fig. S6-Fig. S7 and decision trees for 4-week predictions are shown in Fig. S8-Fig. S10. H: Current hospital occupancy due to COVID-19 (per 100,000 population), A: Rate of weekly new COVID-19 hospital admission averaged over the past 2 weeks (per 100,000 population, dH: Change in weekly new COVID-19 hospitalizations over the past 4 weeks (per 100,000 population), dN: Change in weekly prevalence of novel variant among new infections over the past 4 weeks

Pruned Decision Trees A and B are shown in Fig. 4. Decision Tree A uses surveillance data related to hospital occupancy, the weekly rate of new hospitalizations, and the vaccination coverage to predict whether the hospital occupancy due to COVID-19 would surpass the threshold of 15 per 100,000 population within the next 8 weeks (see Fig. S9 in the Supplement for 4-week projections). Among the features used by this model, three identified as important after optimizing the parameters of the tree: 1) current hospital occupancy due to COVID-19 (per 100,000 population), 2) rate of weekly new COVID-19 hospitalizations averaged over past 2 weeks (per 100,000 population), and 3) change in weekly new COVID-19 hospitalizations over the past 4 weeks (per 100,000 population). Using the validation dataset, we estimated the sensitivity and specificity of this model at 0.936 and 0.833. This decision tree maintains its performance under extreme scenarios that we considered in our sensitivity analyses (Table 3).

**Table 3:** Performance of Decision Trees A and B (Fig. 4) evaluated using 500 simulated trajectories not used for training these models

	Accuracy	Sensitivity	Specificity
Base scenario			
Decision Tree A	0.866	0.936	0.833
Decision Tree B	0.870	0.851	0.879
If non-pharmaceutical measures are removed during the winter and spring of 2022			
Decision Tree A	0.959	0.935	0.962
Decision Tree B	0.961	0.849	0.974
If no novel variant emerges during the winter and spring of 2022			
Decision Tree A	0.887	0.944	0.858
Decision Tree B	0.876	0.821	0.905
Genomic surveillance with small sample size (*N* = 250 tests per week)			
Decision Tree A	0.866	0.936	0.833
Decision Tree B	0.870	0.851	0.879

To illustrate how the Decision Tree A in Fig. 4 can be used by policymakers to predict whether the hospital capacity of 15 per 100,000 population is expected to surpass within 8 weeks, we consider a scenario where the surveillance systems provide the following estimates:

- Current hospital occupancy due to COVID-19 (denoted by H in Fig. 4): 11 per 100,000 population.
- Rate of weekly new COVID-19 hospital admission averaged over the past 2 weeks (denoted by A in Fig. 4): 14 per 100,000 population.

Since H = 11, which is less than 12.88, the condition of the first decision node in satisfied. Hence, we check the condition H ≤ 10.7, which is satisfied. Therefore, we next check the condition A ≤ 13.05. Since A is estimated at 14 from surveillance systems, this classification rule predicts that the hospital capacity of 15 per 100,000 population is expected to be exceeded within 8 weeks. This classification rule would have predicted that hospitalization would stay within the capacity if A was estimated to be less than or equal to 13.05.

In addition to the information available to Decision Tree A, Decision Tree B uses data from genomic surveillance systems to predict whether the hospital occupancy due to COVID-19 would surpass the threshold of 15 per 100,000 population within the next 8 weeks (see Fig. S9 in the Supplement for 4-week projections). The optimized Decision Tree B utilizes four features: change in weekly prevalence of novel variant among new infections over the past 4 weeks in addition to the three features identified as important by Decision Tree A. Using the validation dataset, we estimated the sensitivity and specificity of this model at 0.851 and 0.879. The performance of Decision Tree B also remains robust under extreme scenarios that we considered in our sensitivity analyses (Table 3).

The structure of the proposed decision trees (Fig. 4 and Fig. S6-Fig. S10) reveals that the signals in current hospital occupancy and weekly rate of new hospitalizations are strong enough to accurately predict short- and mid-term surges in hospitalizations despite the substantial uncertainty in factors that determine the local trajectories of COVID-19. The structures of our decision trees also suggest that the estimates of vaccination coverage do not contribute to the accuracy of predictions and the estimates for the prevalence of novel variant among new infections would slightly improve the 8-week predictions only if the hospital occupancy due to COVID-19 is relatively low. Once the rate of hospital occupancy reaches a certain threshold, the transmissibility and virulence of the novel variant is reflected in the hospitalization data; hence, the contributions of estimates for the prevalence of novel variant among new infections would be minimal (Table 3 and Table S13-Table S17 in the Supplement).

## Discussion

We presented a framework to identify simple, easy-to-communicate classification rules that use surveillance data to alert local U.S. policymakers when hospitalizations due to COVID-19 are expected to surpass the local health care capacity within the next 4 or 8 weeks. To identify these decisions rules, we trained classification decision trees using data from thousands of simulated trajectories representing communities with different characteristics that determine the burden of COVID-19, such as population size, age structure, vaccination uptake, effectiveness of mitigating strategies, and the population’s adherence to public health recommendations (Table 2). A main advantage of using simulated trajectories to train decision trees is that simulation model can incorporate complexities, changes, and uncertainties related to the biology of SARS-CoV-2 (e.g., the transmissibility and virulence of novel variants) and additional factors driving local trajectories of COVID-19 (e.g., vaccination rate and the use of mitigating strategies). Therefore, decision trees that are characterized using simulated trajectories that are validated against historical data could be more robust against changes in the data generating process (e.g., due to the spread of a novel pathogen or increase in vaccination rate) and future uncertainties.

There remains substantial uncertainty about how the COVID-19 pandemic will impact local communities in future waves. This is caused by uncertainties in factors such as the effect of seasonality in the transmission of SARS-CoV-2, the duration of infection- and vaccine-induced immunity, the transmissibility, immune evasion, and virulence of novel variants such as omicron and others that may emerge, the vaccine effectiveness against the prevalent and novel variants, and the population’s adherence to public health recommendations during this period (Table 2). Using simulated trajectories distinct from those used to characterize our classification rules, we showed that the accuracy, sensitivity, and specificity of our proposed classification rules are robust to the substantial level of uncertainties surrounding the future of the COVID-19 pandemic at the local level. The performance of these classification rules maintains under extreme scenarios where all non-pharmaceutical interventions are lifted, no novel variant emerges and spreads, and capacity of genomic surveillance is substantially reduced (Table 3 and Table S13-Table S17 in the Supplement)).

Our analysis suggests that classification rules that uses data on current hospital occupancy and the weekly rate of new hospitalizations due to COVID-19 could achieve a high level of sensitivity and specificity in predicting whether hospitalization capacity would be surpassed in the next 4 or 8 weeks. Access to the estimates for vaccination coverage or the prevalence of novel variant among new infections does not markedly improve the performance of these classification rules (Table 3 and Table S13-Table S17 in the Supplement).

Our study has a number of limitations. First, predicting the future trajectory of COVID-19 hospitalizations is challenged by various barriers, some of which are due to uncertainties in epidemic parameters and state variables (Table 2). Although our analysis accounts for these sources of uncertainties, predicting the local trajectories of COVID-19 are further challenged by the unpredictability of population’s behavior and policymakers’ responses to a slowing or speeding pandemic. To minimize the impact of these unpredictable factors, we focused on short- or medium-term (4- or 8-week) predictions. Second, as the data required to develop and evaluate the decision trees considered here are not available in the real world, we had to rely on simulated trajectories to synthetize the datasets needed to train and evaluate our decision trees. As discussed before, the factors driving the COVID-19 pandemic (e.g., public health responses, population behavior and adherence to mitigating strategies, seasonal effects on the transmission of SARS-CoV-2, and vaccination coverage) have continuously changed since the beginning of the pandemic and they will most likely continue to change. Hence, predictive models trained on historical data may not perform well when employed during the upcoming seasons. To mitigate this issue, we used simulated trajectories, which were selected to properly match the historical data and then projected over future months, to produce the datasets needed to train and evaluate our decision trees. This allowed us to account for a wide range of factors, and the uncertainties around them, that will derive the local trajectories of COVID-19 over the medium-term future (Table 2). While we estimated the accuracy, sensitivity, and specificity of each decision model using trajectories not included to train our decision trees, the actual performance of the proposed trees might be different when used in practice. The local policymakers who decide to use the decision trees proposed here are in the ideal position to measure the true accuracy, sensitivity, and specificity of these models using real-world data. Since such data is not currently available, the simulation approach we described here appear to be the only approach at the present to develop and evaluate the proposed classification rules.

Third, our simulation model did not differentiate vaccinated individuals based on the type of vaccine or the number of vaccine doses they have received. However, as none of the proposed decision tree models identified vaccine coverage as an important feature, relaxing this assumption is not expected to change our conclusions. Finally, in addition to surveillance systems we considered in our analysis (Table 1), data from other surveillance systems may also be available and used to provide information about different aspects of the pandemic. This may include genomic surveillance at hospitals to estimate the proportion of hospitalizations that are due to novel variants or potential vaccine-escape SARS-CoV-2 variants [26, 28], and seroprevalence surveillance to estimate the percentage of populations who have antibodies against SARS-CoV-2 [24]. While including data from these sources could improve the performance of classification rules developed here, these sources are not always avilable at granular geographic regions.

Since the beginning of the COVID-19 pandemic, numerous models have been developed to predict the future trajectory of the pandemic (e.g., COVID-19 Forecast Hub [3] or the IHME COVID-19 Forecasting Model [4]). The results of these predictive models are usually available at the national or state levels. Therefore, the usefulness of these models for local policymakers are limited since the local trajectory of the pandemic could be substantially different from the predictions made at the larger geographic regions. The simple, easy-to-communicate classification rules we characterized in this study could be used to alert local policymakers when the hospital occupancy due to COVID-19 is to exceed the local hospital capacity.

While we validated these classification rules using trajectories under various scenarios, the true performance of these classification rules is to be seen. If the true accuracy, sensitivity, and specificity of the proposed classification rules turn out to be similar to what we estimated using simulated trajectories, the work presented here offers a novel and innovative approach to assist local policymakers in responding to future pandemics when real-word data to inform predictive and simulation models are scarce or not yet available. The main utility of classification rules characterized here is not derived from the ability to predict the hospitalizations surges with 100% accuracy. Instead, the proposed framework offers a principled approach to identify communities with healthcare systems at risk being strained. Finally, the framework described here (Fig. 1) allows for updating these classification rules in response to major changes in the properties of the epidemic systems (e.g., the emergence of new variants or the widespread availability of effective antivirals) and the latest evidence regarding the key epidemic parameters. This ensures that the characterized classification rules are consistent with the latest evidence.

## Supplementary Information

Below is the link to the electronic supplementary material.

Supplementary file1 (PDF 2270 KB)

## Data Availability

All data and code to reproduce our analysis is available at https://github.com/yaesoubilab/COVIDRiskClassificationRules-HCMS.
