# Supplementary material for: Generating simple classification rules to predict local surges in COVID-19 hospitalizations
Source: Health Care Manag Sci. 2023 Jan 24;26(2):301–12. doi: 10.1007/s10729-023-09629-4 (PMC9872755; doi:10.1007/s10729-023-09629-4)
Supplement: Supplementary file 1 — Supplementary file1 (PDF 2270 KB) [file 10729_2023_9629_MOESM1_ESM.pdf]

Supplementary Material: Additional Details on Simulation and Predictive Models

S1 Initialization of the simulation model

The model is initialized with a random number of susceptible individuals, drawn from the uniform distribution [250,000, 1,250,00]. The susceptible individuals are then randomly assigned according to the age groups 0-4, 5-12, 13-17, 18-29, 30-49, 50-64, 65-74, and 75+ according to the U.S. age-distribution (respectively, 6.0%, 10.0%, 6.4%, 16.3%, 25.7%, 19.2%, 9.6%, and 6.9%) [1]. A number of infectious individuals, determined based on a random draw from the discrete-uniform (1, 5), are imported to Infectious compartments according to a Multinominal distribution with probabilities equal to the U.S. age distribution.

S2 Transmission model

S2.1 Simulation framework

To construct the model, we introduce the following notation:

- $i \in \{1, 2, 3, 4, 5, 6, 7, 8\}$ index of age groups {0-4, 5-12, 13-17, 18-29, 30-49, 50-64, 65-74, 75+}.
- $k \in \{1,2,3\}$ index of infection profile {infected with the ancestral strain, infected with the delta variant, infected with the novel variant}.
- $v \in \{0,1\}$ index of vaccination status
- t: epidemic time (in year).
- $N_i(t)$: number of individuals in age group i at time t.
- $S_i(t)$: number of susceptible individuals in age group i at time t.
- $V_i(t)$: number of vaccinated individuals in age group i at time t.
- $E_{i,k,v}(t)$: number of exposed individuals in age group i and with infection profile k and vaccination status v at time t.
- $I_{i,k,v}(t)$: number of infectious individuals in age group i and with infection profile k and vaccination status v at time t.
- $H_{i,k,v}(t)$: number of hospitalized individuals in age group i and with infection profile k and vaccination status v at time t.
- $R_{i,k,v}(t)$: number of recovered individuals in age group i and with infection profile k and vaccination status v at time t.

The state of the epidemic at any given time t can be identified by a discrete-time Markov chain $\{(S_i(t), V_i(t), E_{i,k,v}(t), I_{i,k,v}(t), H_{i,k,v}(t), R_{i,k,v}(t), i \in \{1,2, \dots ,8\}, k \in \{1, 2, 3\}, v \in \{0,1\}: t = 0, \Delta t, 2\Delta t, 3\Delta t, \dots\}$, where Δt is the time-step of the simulation (e.g., $\Delta t = 1$ day). To generate epidemic trajectories for this model, we use Monte Carlo simulation to sample from this Markov chain using the following approach. Consider a

particular compartment Z in which members depart due to J events each of which is occurring at the rate $\mu_j, j \in \{1,2,\dots,J\}$. For example, members of Susceptible compartment may leave due to 1) infection with the ancestral strain, 2) infection with the delta variant, 3) infection with the novel variant, or 4) vaccination (i.e., $J = 4$) (see Fig. 2). If the number of individuals in compartment Z at time t is $Z(t)$, then the number of individuals that leave this compartment due to events $j \in \{1,2,\dots,J\}$ follows a multinomial distribution with total counts of $Z(t)$ and probabilities $(p_0, p_1, p_2, \dots p_J)$, where $p_0 = e^{-\sum_{j=1}^{J} \mu_j \Delta t}$ is the probability of not leaving the compartment Z during $[t, t + \Delta t]$, and $p_j = \frac{\mu_j}{\sum_{j=1}^{J} \mu_j \Delta t}(1 - p_0)$ is the probability of leaving the compartment Z during $[t, t + \Delta t]$ due to the event $j \in \{1,2,\dots,J\}$.

To identify the new epidemic state at the next time step, we first sample from the multinomial distributions associated to each compartment and then use these realizations to calculate the new epidemic state given the current epidemic state. The events that drive the epidemic are represented by black arrows in Fig. 2. For example, the number of susceptibles in age group i at time $t + \Delta t$ can be calculated as:

$$
\begin{aligned}
&S_i(t+\Delta t) = S_i(t) \\
&- \text{new infections with ancestral strain in age group } i \\
&- \text{new infections with the delta variant in age group } i \\
&- \text{new infections with novel variant in age group } i \\
&- \text{new vaccinations in age group } i \\
&+ \text{members losing infection-induced immunity in age group } i \\
&+ \text{members losing vaccine-induced immunity in age group } i.
\end{aligned}
$$

S2.2 Rate of infection

For susceptible members in age group i, we calculate the rate of infection with ancestral strain at time t as:

$$\mathcal{F}_{i,0}(t) = \beta(t) \sum_j \lambda_{i,j} \left(\frac{I_{j,0,0}(t)}{N_j(t)} + \frac{I_{j,0,1}(t)}{N_j(t)} \eta_{0,1} \right), \tag{1}$$

where $\beta(t)$ is the transmission parameter for the ancestral strain at time t, $\lambda_{i,j}$ is the daily rate at which an average individual in age group i contact with individuals in age group j (see below for how $\lambda_{i,j}$ is estimated), and $\eta_{0,1} \in [0,1]$ is the ratio of infectiousness for a vaccinated individual to an unvaccinated one who are infected with the ancestral strain.

For susceptible members in age group i, we calculate the rate of infection with the delta variant and a novel variant at time t as:

$$\mathcal{F}_{i,1}(t) = \beta(t) \sum_j \lambda_{i,j} \eta_1 \left(\frac{I_{j,1,0}(t)}{N_j(t)} + \frac{I_{j,1,1}(t)}{N_j(t)} \eta_{1,1} \right), \tag{2}$$

$$\mathcal{F}_{i,2}(t) = \beta(t) \sum_j \lambda_{i,j} \eta_2 \left(\frac{I_{j,2,0}(t)}{N_j(t)} + \frac{I_{j,2,1}(t)}{N_j(t)} \eta_{2,1} \right), \tag{3}$$

Where $\eta_1 \geq 0$ and $\eta_2 \geq 0$ are the ratio of infectiousness for the delta and the novel variant to the ancestral strain, and $\eta_{1,1} \in [0,1]$ and $\eta_{2,1} \in [0,1]$ are the ratio of infectiousness for a vaccinated individual who is infected with the delta variant and the novel variant to the unvaccinated individual who is infected with the ancestral strain.

For vaccinated members in age group i, we calculate the rate of infection with variant $k \in \{1,2,3\}$ at time t as:

$$\mathcal{F}_{i,k}^{V}(t) = (1-\gamma_k)\mathcal{F}_{i,k}(t), \quad (4)$$

where $\gamma_k \in [0,1]$ is the effectiveness of vaccination in providing immunity against infection with the variant k.

To capture the effect of seasonal changes on the transmission of SARS-CoV-2, we allow the transmission parameter $\beta(t)$ to vary over time according to:

$$\beta(t) = a_0 + a_1 \cos 2\pi(t-\phi)\,. \quad (5)$$

Here, a_0 is the baseline transmissibility which is not influenced by the seasonality effect, and the parameter a_1 represents the maximum magnitude of seasonality effect during a year. The phase parameter ϕ determines when the effect of seasonality reaches its maximum or minimum and is included to provide additional flexibility in the modeling of the seasonality effect. We determined that a_0, a_1, and ϕ by random draws from uniform distributions $U[0.75, 1.25]$, $U[0, 0.5]$, and $U[-0.25, 0]$. See Fig. S4 for how $\beta(t)$ behaves for different values of a_0, a_1, and ϕ.

S2.3 Daily contact rate

Let $\hat{\lambda}_{i',j'}$ denote the average number of contacts per day an individual in age group j' makes with individuals in age group i', as estimated by Prem et al. [2] for the U.S. population. Age groups considered in this survey include $\hat{\mathcal{A}}$ = {0-4, 5-9, 10-14, 5-19, 20-24, 25-29, 30-34, 35-39, 40-44, 45-49, 50-54, 55-59, 60-64, 65-69, 70, 75+}.

Since our model only includes age groups $\mathcal{A}$ = {0-4, 5-12, 13-17, 18-29, 30-49, 50-64, 65-74, 75+}, we use estimates $\hat{\lambda}_{i',j'}$, $i', j' \in \hat{\mathcal{A}}$, to calculate the contact matrix $[\lambda_{i,j}]$, $i, j \in \mathcal{A}$ to be used in our model. To this end, we make the following assumption:

1. Contacts *within* an age group are uniformly distributed; that is, a contact with an average member in age group {0-4}, for example, is equally likely to be with someone of age 0, 1, 2, 3, or 4.
2. If in estimating $\lambda_{i,j}$, the age group $j \in \mathcal{A}$ includes multiple age groups from $\hat{\mathcal{A}}$ (say j_1', j_2', ...), then $\lambda_{i,j} = \sum_{j' \in \{j_1', j_2', \dots\}} \hat{\lambda}_{i',j'}$. For example, $\lambda_{\{35-39\},\{64-74\}} = \hat{\lambda}_{\{35-39\},\{64-69\}} + \hat{\lambda}_{\{35-39\},\{70-74\}}$.
3. If in estimating $\lambda_{i,j}$, age group $i \in \mathcal{A}$ overlaps with multiple age groups from $\hat{\mathcal{A}}$ (say i_1', i_2', ...), the daily contact $\lambda_{i,j}$ will be the average of $\lambda_{i_1',j}$, $\lambda_{i_2',j}$, ... For example, $\lambda_{\{30-49\},\{0-4\}} = (\hat{\lambda}_{\{30-34\},\{0-4\}} + \hat{\lambda}_{\{35-39\},\{0-4\}} + \hat{\lambda}_{\{40-44\},\{0-4\}} + \hat{\lambda}_{\{45-49\},\{0-4\}})/4$.

Following the approach of Medlock et al. [3], we then ensured that the number of contacts between age groups is symmetric (i.e. $\varpi_i \lambda_{i,j} = \varpi_j \lambda_{j,i}$, where ϖ_i is the size of age group i), by using $\bar{\lambda}_{i,j} = \frac{1}{2\varpi_i}(\varpi_i \lambda_{i,j} + \varpi_j \lambda_{j,i})$. The contact rate matrix $[\lambda_{i,j}]$ used in our model is shown in Table S1.

Table S1: Average daily number of contacts between different age groups used in our model

Age Groups	**0-4**	**5-12**	**13-17**	**18-29**	**30-49**	**50-64**	**65-74**	**75+**
**0-4**	2.598	1.312	0.316	1.146	2.551	1.080	0.258	0.141
**5-12**	0.786	6.398	2.266	1.069	3.280	1.306	0.365	0.207
**13-17**	0.295	3.533	6.294	5.178	3.624	1.687	0.296	0.216
**18-29**	0.419	0.652	2.026	6.492	4.244	1.944	0.230	0.104
**30-49**	0.591	1.268	0.899	2.691	6.790	2.366	0.412	0.195
**50-64**	0.336	0.678	0.562	1.655	3.176	2.944	0.476	0.173
**65-74**	0.161	0.379	0.197	0.392	1.105	0.951	1.144	0.253
**75+**	0.122	0.299	0.200	0.247	0.729	0.482	0.352	0.396

S2.4 Effectiveness of control measures

We assume that control measures went into effect whenever the rate of hospital occupancy due to COVID-19 exceeded the threshold T_1 and were lifted whenever this rate dropped below the second threshold T_2 [4]. These thresholds differ across simulated trajectories and were determined by random draws from uniform distributions $U(1, 50)$ and $U(1, 50)$ per 100,000 population. We assume that the impact of control measures ($\alpha(h)$) in reducing the effective reproductive number varies as a function of hospital occupancy due to COVID-19 (h) according to the sigmoid function $\alpha(h) = \frac{\bar{\alpha}}{1+e^{-4h/\bar{h}}}$, where $\bar{\alpha}$ is the maximum impact of control measures and $\bar{h}$ is the maximum hospital capacity that could be allocated for COVID-19 patients (Fig. S3). We determined $\bar{\alpha}$ and $\bar{h}$ by random draws from uniform distributions $U(50\%, 85\%)$ [4-6] and $U(5, 15)$ per 100,000 population [7].

S3 Training decision tree models

We used the scikit-learn package to train decision tree models [8]. Before training the models, we created a balanced dataset where the number of observations for which the hospital occupancy passed the specified threshold is equal to the number of observations for which this event didn't occur. To avoid overfitting, we used a minimal cost-complexity pruning approach [9], where we determined the complexity parameter using 10-fold cross-validation to maximize the model accuracy (defined as the fraction of correct predictions) [10]. For each decision tree model, we chose the optimal value of the complexity parameter from {0, 0.005, 0.01, 0.015, 0.02, …, 0.1}.

S4 Supplementary Tables

Table S2: Model parameters related to dominant strain

Parameter	Prior Distribution#	95% Percentile Interval†	Source
R_0	Beta (mean=2.5, SD=0.75, min=1.5, max=4)	(1.52, 3.85)	[11, 12]
Delay from infection until becoming infectious (days)	Beta (mean=5, SD=0.5, min=4, max=6)	(4.06, 5.96)	[13]
Duration of infectiousness (days)	Beta (mean=4, SD=0.5, min=2, max=8)	(2.05, 7.22)	[14, 15]
Duration of hospitalization (days)	Beta (mean=12, SD=1, min=7, max=17)	(10.06, 13.94)	[16]
Duration of infection-induced immunity (years)	Beta (mean=1, SD=0.25, min=0.25, max=1.5)	(0.493, 1.42)	[6, 17]
Relative probability of hospitalization for the dominant strain			⁋
0-4	Gamma (mean=0.5, SD=0.1)		
5-12	Gamma (mean=0.5, SD=0.1)		
13-17	Gamma (mean=0.25, SD=0.05)		
18-29	Reference group		
30-49	Gamma (mean=2.0, SD=0.5)		
50-64	Gamma (mean=4.0, SD=1.0)		
65-74	Gamma (mean=6.0, SD=1.5)		
75+	Gamma (mean=10.8, SD=2.7)		
Probability of hospitalization for age group 18-29 if infected	Uniform (0.0005, 0.005)		Assumption
Probability death if requiring hospitalization (%)			[18]
0-4	Beta (mean=0.2, SD=0.05)		
5-12	Beta (mean=0.2, SD=0.05)		
13-17	Beta (mean=0.2, SD=0.05)		
18-29	Beta (mean=2.6, SD=0.65)		
30-49	Beta (mean=2.6, SD=0.65)		
50-64	Beta (mean=7.9, SD=1.98)		
65-74	Beta (mean=14.1, SD=3.53)		
75+	Beta (mean=20.9, SD=5.23)		
Weekly rate of importation of new infection	5		Assumption

SD: Standard deviation

† 95% Percentile Interval is defined as [2.5th percentile, 97.5th percentile] of the assumed probability distribution.

⁋ Risk for COVID-19 Infection, Hospitalization, and Death by Age Group (https://www.cdc.gov/coronavirus/2019-ncov/covid-data/investigations-discovery/hospitalization-death-by-age.html).

Table S3: Model parameters related to novel variant

Parameter	Distribution#	Source
Weekly importation rate of the delta variant ($\gamma(t) = b_{max}/(1 + e^{-b(t-t_{mid})})$, see Fig. S1)		Assumption
b	Beta (mean=7, SD=0.5, min=5, max=9)	
b_{max}	Uniform (2, 5)	
t_{mid}	Uniform (1.5, 1.75)	
Importation rate of the novel variant ($\gamma(t) = b_{max}/(1 + e^{-b(t-t_{mid})})$, see Fig. S1)		Assumption
b	Beta (mean=7, SD=0.5, min=5, max=9)	
b_{max}	Uniform (0, 5)	
t_{mid}	Uniform (1.5, 2)	
Delay from infection until becoming infectious (days)	Same distribution as dominant stain in Table S2	Assumption
Duration of hospitalization (days)	Same distribution as dominant stain in Table S2	Assumption
Duration of infection-induced immunity (years)	Same distribution as dominant stain in Table S2	Assumption
Ratio of transmissibility to that of the dominant strain	Uniform (1, 2)	[19, 20]
Ratio of the duration of infectiousness for the novel strain to that of the dominant strain	Uniform (0.5, 2)	Assumption
Ratio of the probability of requiring hospitalization to that of the dominant strain	Uniform (0.5, 3)	[21-23]
Probability death if requiring hospitalization (%)	The same as dominant stain in Table S2.	Assumption
Effectiveness of the recent recovery from infection with the dominant strain in providing immunity against infection with the novel strain	Uniform (50%, 100%)	Assumption

Table S4: Model parameters related to vaccinated individual who are infected with the novel variant

Parameter	Distribution#	Source
Delay from infection until becoming infectious (days)	Same distribution as dominant stain in Table S2	Assumption
Duration of hospitalization (days)	Same distribution as dominant stain in Table S2	Assumption
Duration of infection-induced immunity (years)	Same distribution as dominant stain in Table S2	Assumption
Vaccine effectiveness against novel variant		[24]
Ratio of susceptibility of a vaccinated individual that of an unvaccinated individual	Uniform (0, 1)	
Ratio of transmissibility to that of the dominant strain	Uniform (0, 0.5)	
Ratio of duration of infectiousness for the vaccinated individuals to that of the dominant strain	Uniform (0, 0.5)	
Ratio of the probability of requiring hospitalization for the vaccinated individuals to that of the dominant strain	Uniform (0, 0.5)	
Probability death if requiring hospitalization (%)	The same as dominant stain in Table S2.	

Table S5: Model parameters related to vaccine and vaccination

Parameter	Distribution#	Source
Duration of vaccine-induced immunity, accounting for booster doses (years)	Uniform (0.5, 2.5)	[6]
Increase in the duration of infection-induced immunity after vaccination	Uniform (0%, 50%)	Assumption
Vaccine effectiveness against infection		Assumption based on [20]
For dominant strain	Uniform (0%, 100%)	
For novel strain	Uniform (0%, 100%)	
Vaccine effectiveness against hospitalization		Assumption based on [20, 25]
For dominant strain	Uniform (85%, 100%)	
For novel strain	Uniform (0%, 100%)	
Vaccine effectiveness in reducing infectiousness		Assumption based on [26]
For dominant strain	Uniform (25%, 75%)	
For novel strain	Uniform (25%, 75%)	
Annual vaccination rate $\boldsymbol{v_k(t)} = b_{max} + (b_{max} - b_{min})/(1 + e^{-b(t-t_{mid}-t_{min})})$ if $t \geq t_{min}$ (see Fig. S2)		Assumption to match the estimated vaccination coverage (Table S8)
b	Uniform (-20, -10)	
b_{min}	0	
t_{mid}	Uniform (0.25, 0.75)	
b_{max}	Uniform (2, 3)	
b_{max}	Uniform (0.25, 0.75)	
5-12	Uniform (1, 3)	
13-17	Uniform (1, 3)	
18-29	Uniform (1, 3)	
30-49	Uniform (1, 3)	
50-64	Uniform (2, 4)	
65-74	Uniform (3, 5)	
75+	Uniform (2, 4)	
t_{min}		
5-12	Uniform (1.6, 1.8)	
13-17	Uniform (1.0, 1.4)	
18-29	Uniform (0.85, 1.25)	
30-49	Uniform (0.85, 1.25)	
50-64	Uniform (0.85, 1.25)	
65-74	Uniform (0.75, 1.15)	
75+	Uniform (0.7, 1.1)	

SD: Standard deviation
† 95% Percentile Interval is defined as [2.5th percentile, 97.5th percentile] of the assumed probability distribution.

Table S6: Model parameters related to mitigating strategies

Parameter	Distribution[#]	Source
Effectiveness of control measures ($\alpha(h) = \frac{\bar{\alpha}}{1+e^{-4h/\bar{h}}}$)		
Maximum effectiveness of control measures ($\bar{\alpha}$)	$U(50\%, 85\%)$	[4-6]
Maximum hospitalization capacity that could be allocated to patients with COVDI-19, per 100,000 population ($\bar{h}$)	$U(5, 15)$	[7]

Table S7: Summary of COVID-NET hospitalization data to inform selection of simulated trajectories.

**Weekly hospitalization rate (per 100,000 population) between March 1, 2010 and November 27, 2021** (source: https://gis.cdc.gov/grasp/COVIDNet/COVID19_3.html)			
		Minimum value achieved in all States included in COVID-NET	Maximum value achieved in all States included in COVID-NET
Overall		8.9	51.2
**Cumulative hospitalization rate (per 100,000 population) between March 1, 2010 and November 27, 2021** (source: https://gis.cdc.gov/grasp/COVIDNet/COVID19_3.html)			
	Cumulative Hospitalization Rate	Minimum value among all States included in COVID-NET	Maximum value among all States included in COVID-NET
Overall	768.0	301.7	1050.3
0-4	100.2	50.6	161.2
5-11	33.7	17.4	46.6
12-17	81.4	43.5	129.7
18-29	298.8	85.0	508.0
30-49	601.5	179.3	897.0
50-64	1124.2	449.0	1490.7
65-74	1590.9	750.1	2356.8
75+	2973.2	1553.9	4313.6

Table S8: Estimated vaccine coverage (i.e., the percentage of the population that are fully vaccinated) over time by age (source: https://covid.cdc.gov/covid-data-tracker/#vaccination-demographics-trends)

**Date**	**Overall**	**0-4**	**5-12**	**13-17**	**18-29**	**30-49**	**50-64**	**65-75**	**75+**
Jan 1, 2021	0.01%	0.00%	0.00%	0.00%	0.01%	0.02%	0.02%	0.02%	0.01%
Jan 26, 2021 (week 4)	1.72%	0.00%	0.00%	0.02%	1.53%	2.63%	2.56%	1.68%	1.94%
Feb 26, 2021 (week 8)	9.39%	0.00%	0.01%	0.13%	5.39%	8.51%	9.88%	21.22%	32.80%
Mar 26, 2021 (week 12)	18.79%	0.00%	0.01%	0.36%	9.34%	14.58%	20.31%	53.66%	60.95%
Apr 23, 2021 (week 16)	33.75%	0.00%	0.01%	2.09%	21.65%	30.79%	47.94%	76.76%	75.76%
May 21, 2021 (week 20)	45.70%	0.00%	0.02%	9.14%	38.53%	47.83%	64.78%	83.93%	80.63%
June 18, 2021 (week 24)	51.65%	0.00%	0.34%	26.34%	46.26%	54.87%	70.60%	86.92%	82.75%
July 16, 2021 (week 28)	54.31%	0.00%	0.44%	34.16%	49.93%	57.99%	73.02%	88.27%	83.70%
Aug 13, 2021 (week 32)	56.46%	0.00%	0.45%	39.98%	52.94%	60.55%	75.09%	89.45%	84.51%
Sep 10, 2021 (week 36)	59.74%	0.00%	0.45%	48.54%	57.25%	64.70%	78.22%	91.22%	85.72%
Oct 8, 2021 (week 40)	62.44%	0.00%	0.45%	53.62%	61.25%	68.41%	80.69%	92.83%	86.91%
Nov 5, 2021 (week 44)	64.13%	0.00%	0.46%	56.01%	63.61%	70.66%	82.29%	94.32%	88.24%
Dec 3, 2021 (week 48)	65.74%	0.00%	4.63%	57.52%	65.21%	72.26%	83.57%	95.42%	89.24%

We note that the age groups in our model is different from the age groups of COVID Data Tracker. To estimate the vaccine coverage for age-group 5-12, we divide the total number of vaccinations by 2/3 the population size of in age groups <12. To estimate the vaccine coverage for age group 13-17, we divide the number of vaccinations in age group 12-15 and 16-17 by the population sizes of the age groups. To estimate the vaccine coverage for age group 18-29, we divided the total number of vaccinations in age group 18-24 and 1/3 in age group 25-39 by the same proportions of the population sizes of the age groups. To estimate the vaccine coverage for age group 30-49, we divided 2/3 of vaccinations in age group 25-39 and the total number in age group 40-49 by the same proportions of the population sizes of the age groups.

Table S9: Estimated vaccination coverage (i.e., the percentage of the population that are fully vaccinated) (source: https://github.com/govex/COVID-19/tree/master/data_tables/vaccine_data)

Rate of vaccination on December 07, 2021			
	Mean	Minimum value achieved in all States	Maximum value achieved in all State
Overall	59.83%	46.21%	80.64%

Table S10: Percentage of cases due to delta variant https://covid.cdc.gov/covid-data-tracker/#variant-proportions

Last Day of 2-week Sequencing	USA	Minimum value observed among 10 U.S. regions	Maximum value observed among 10 U.S. regions
4/10/2021	0.1%	0.0%	0.3%
4/24/2021	0.6%	0.3%	1.2%
5/8/2021	1.4%	0.8%	2.4%
5/15/2021	2.4%	1.2%	7.5%
5/22/2021	3.8%	2.1%	13.9%
5/29/2021	7.0%	2.3%	23.6%
6/5/2021	14.1%	5.1%	44.7%
6/12/2021	26.1%	4.9%	70.6%
6/19/2021	37.2%	16.3%	77.7%
6/26/2021	53.6%	28.1%	87.8%
7/3/2021	69.4%	51.9%	93.0%
7/10/2021	82.2%	70.9%	95.9%
7/17/2021	87.6%	82.6%	95.9%
7/24/2021	94.4%	93.1%	97.0%
7/31/2021	96.8%	93.8%	99.0%
8/7/2021	97.9%	96.4%	99.6%
8/14/2021	98.8%	97.7%	99.8%

Table S11: Correlations between the features defined in Table 1 and the event that hospital capacity would surpass the specified thresholds within 8 weeks.

		**Threshold for Hospital Occupancy (per 100,000 population)**					
		**10**		**15**		**20**	
**Surveillance**	**Features used for prediction**	**Corr**	***p*-value**	**Corr**	***p*-value**	**Corr**	***p*-value**
Rate of hospital occupancy	Current value	0.507	<0.001	0.613	<0.001	0.678	<0.001
Weekly rate of new hospitalizations	Average over the past 2 weeks	0.464	<0.001	0.549	<0.001	0.595	<0.001
	Average change during the past 4 weeks	0.074	<0.001	0.114	<0.001	0.151	<0.001
Vaccination coverage	Cumulative value	-0.083	<0.001	-0.11	<0.001	-0.118	<0.001
Prevalence of novel variant among new infections	Average over the past 2 weeks	0.023	0.050	0.028	0.02	0.011	0.348
	Average change during the past 4 weeks	0.032	0.008	0.049	<0.001	0.035	0.003

Table S12: Correlations between the features defined in Table 1 and the event that hospital capacity would surpass the specified thresholds within 4 weeks.

		**Threshold for Hospital Occupancy (per 100,000 population)**					
		**10**		**15**		**20**	
**Surveillance**	**Features used for prediction**	**Corr**	***p*-value**	**Corr**	***p*-value**	**Corr**	***p*-value**
Rate of hospital occupancy	Current value	0.598	<0.001	0.71	<0.001	0.767	<0.001
Weekly rate of new hospitalizations	Average over the past 2 weeks	0.552	<0.001	0.639	<0.001	0.67	<0.001
	Average change during the past 4 weeks	0.112	<0.001	0.167	<0.001	0.226	<0.001
Vaccination coverage	Cumulative value	-0.071	<0.001	-0.094	<0.001	-0.098	<0.001
Prevalence of novel variant among new infections	Average over the past 2 weeks	0.025	0.019	0.026	0.015	0.029	0.006
	Average change during the past 4 weeks	0.035	0.001	0.043	<0.001	0.047	<0.001

Table S13: Performance of Decision Trees A and B (Fig. S6; 8-week prediction for the hospital occupancy threshold of 10 per 100,000 population) evaluated using 500 simulated trajectories not used for training these models.

	**Accuracy**	**Sensitivity**	**Specificity**
**Base scenario**			
Decision Tree A	0.918	0.871	0.928
Decision Tree B	0.917	0.785	0.947
**If non-pharmaceutical measures are removed during the winter and spring of 2022**			
Decision Tree A	0.965	0.819	0.974
Decision Tree B	0.969	0.733	0.984
**If no novel variant emerges during the winter and spring of 2022**			
Decision Tree A	0.911	0.852	0.924
Decision Tree B	0.915	0.834	0.933
**Genomic surveillance with small sample size (*N*=250 tests per week)**			
Decision Tree A	0.918	0.871	0.928
Decision Tree B	0.917	0.782	0.947

Table S14: Performance of Decision Trees A and B (Fig. S7; 8-week prediction for the hospital occupancy threshold of 20 per 100,000 population) evaluated using 500 simulated trajectories not used for training these models.

	**Accuracy**	**Sensitivity**	**Specificity**
**Base scenario**			
Decision Tree A	0.858	0.943	0.794
Decision Tree B	0.863	0.903	0.833
**If non-pharmaceutical measures are removed during the winter and spring of 2022**			
Decision Tree A	0.941	0.875	0.954
Decision Tree B	0.943	0.822	0.967
**If no novel variant emerges during the winter and spring of 2022**			
Decision Tree A	0.874	0.919	0.83
Decision Tree B	0.874	0.886	0.863
**Genomic surveillance with small sample size (*N*=250 tests per week)**			
Decision Tree A	0.858	0.943	0.794
Decision Tree B	0.863	0.903	0.833

Table S15: Performance of Decision Trees A and B (Fig. S8; 4-week prediction for the hospital occupancy threshold of 10 per 100,000 population) evaluated using 500 simulated trajectories not used for training these models.

	**Accuracy**	**Sensitivity**	**Specificity**
**Base scenario**			
Decision Tree A	0.946	0.930	0.952
Decision Tree B	0.946	0.930	0.952
**If non-pharmaceutical measures are removed during the winter and spring of 2022**			
Decision Tree A	0.98	0.933	0.985
Decision Tree B	0.98	0.933	0.985
**If no novel variant emerges during the winter and spring of 2022**			
Decision Tree A	0.952	0.955	0.952
Decision Tree B	0.952	0.955	0.952
**Genomic surveillance with small sample size (*N*=250 tests per week)**			
Decision Tree A	0.946	0.930	0.952
Decision Tree B	0.946	0.930	0.952

Table S16: Performance of Decision Trees A and B (Fig. S9; 4-week prediction for the hospital occupancy threshold of 15 per 100,000 population) evaluated using 500 simulated trajectories not used for training these models.

	**Accuracy**	**Sensitivity**	**Specificity**
**Base scenario**			
Decision Tree A	0.934	0.93	0.937
Decision Tree B	0.934	0.93	0.937
**If non-pharmaceutical measures are removed during the winter and spring of 2022**			
Decision Tree A	0.976	0.931	0.986
Decision Tree B	0.976	0.931	0.986
**If no novel variant emerges during the winter and spring of 2022**			
Decision Tree A	0.941	0.93	0.949
Decision Tree B	0.941	0.93	0.949
**Genomic surveillance with small sample size (*N*=250 tests per week)**			
Decision Tree A	0.934	0.93	0.937
Decision Tree B	0.934	0.93	0.937

Table S17: Performance of Decision Trees A and B (Fig. S10; 4-week prediction for the hospital occupancy threshold of 20 per 100,000 population) evaluated using 500 simulated trajectories not used for training these models.

	**Accuracy**	**Sensitivity**	**Specificity**
**Base scenario**			
Decision Tree A	0.924	0.967	0.873
Decision Tree B	0.924	0.967	0.873
**If non-pharmaceutical measures are removed during the winter and spring of 2022**			
Decision Tree A	0.966	0.948	0.973
Decision Tree B	0.966	0.948	0.973
**If no novel variant emerges during the winter and spring of 2022**			
Decision Tree A	0.932	0.962	0.889
Decision Tree B	0.932	0.962	0.889
**Genomic surveillance with small sample size (*N*=250 tests per week)**			
Decision Tree A	0.924	0.967	0.873
Decision Tree B	0.924	0.967	0.873

S5 Supplementary Figures

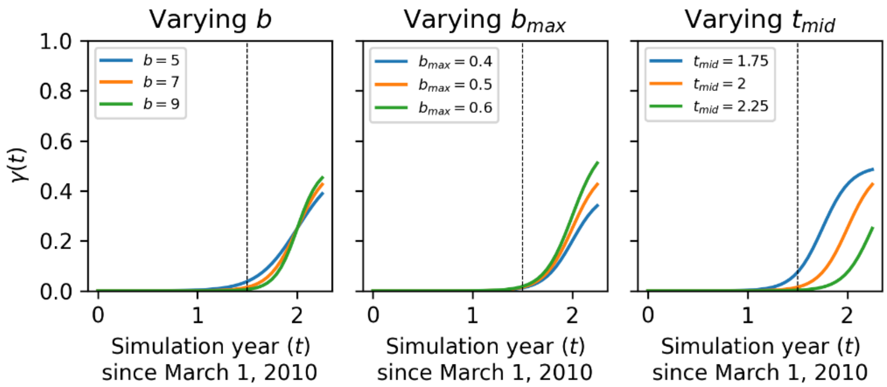

Fig. S1: Probability that an imported case is infected with the novel strain. We assumed that this probability increases according to $\boldsymbol{\gamma(t) = b_{max}/(1 + e^{-b(t-t_{mid})})}$. The probability distributions assumed for $\boldsymbol{b_{max}}$, $\boldsymbol{b}$, and $\boldsymbol{t_{mid}}$ are listed in Table S3. The vertical dotted lines marks September 1, 2021.

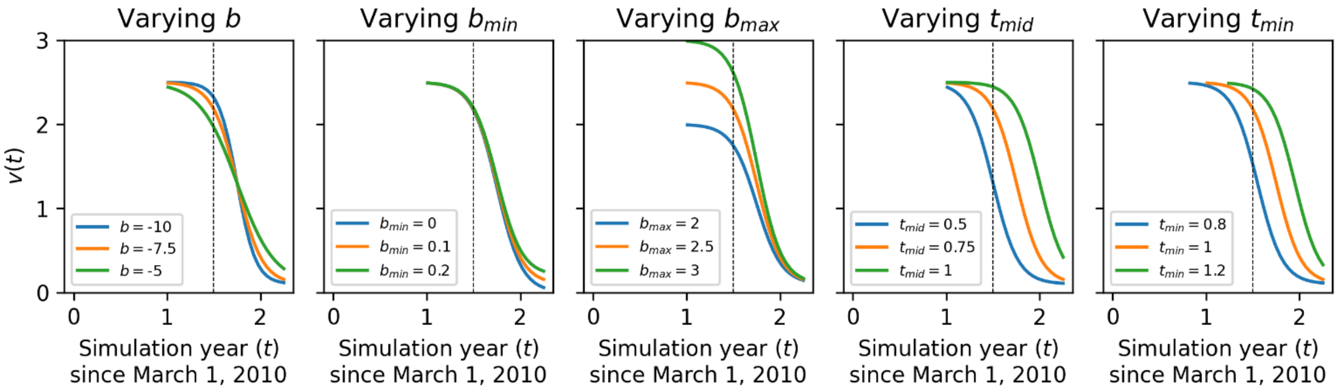

Fig. S2: The rate at which population is getting vaccinated. We assumed that vaccination rate in an age group decreases according to $\boldsymbol{v(t) = b_{max} + (b_{max} - b_{min})/(1 + e^{-b(t-t_{mid}-t_{min})})}$ if $\boldsymbol{t \geq t_{min}}$. The probability distributions assumed for $\boldsymbol{b_{max}}$, $\boldsymbol{b_{min}}$, $\boldsymbol{b}$, $\boldsymbol{t_{mid}}$, and $\boldsymbol{t_{min}}$ are listed in Table S5. The vertical dotted lines marks September 1, 2021.

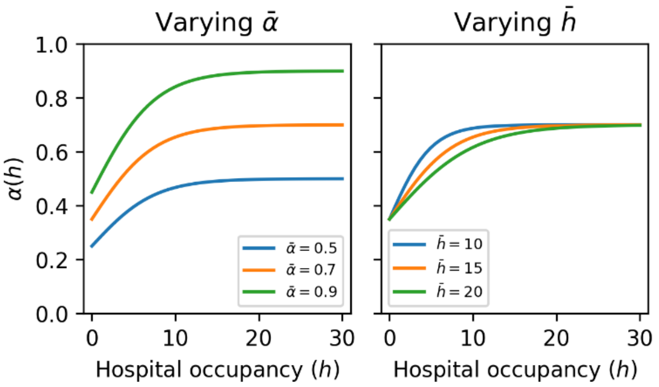

Fig. S3: The effectiveness of control measures as the function of hospital occupancy. We assumed that the effectiveness of control strategies increases with the hospital occupancy according to $\boldsymbol{\alpha(h) = \frac{\bar{\alpha}}{1+e^{-4h/\bar{h}}}}$. The probability distributions assumed for $\bar{\boldsymbol{\alpha}}$ and $\bar{\boldsymbol{h}}$ are listed in Table S6.

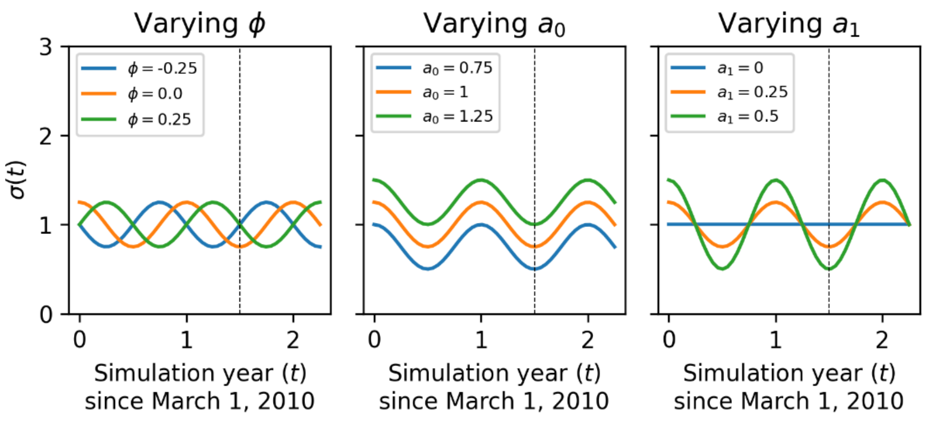

Fig. S4: Modeling seasonality effect according to the function $\boldsymbol{\beta(t) = a_0 + a_1 \cos 2\pi(t - \phi)}$. The vertical dotted lines marks September 1, 2021.

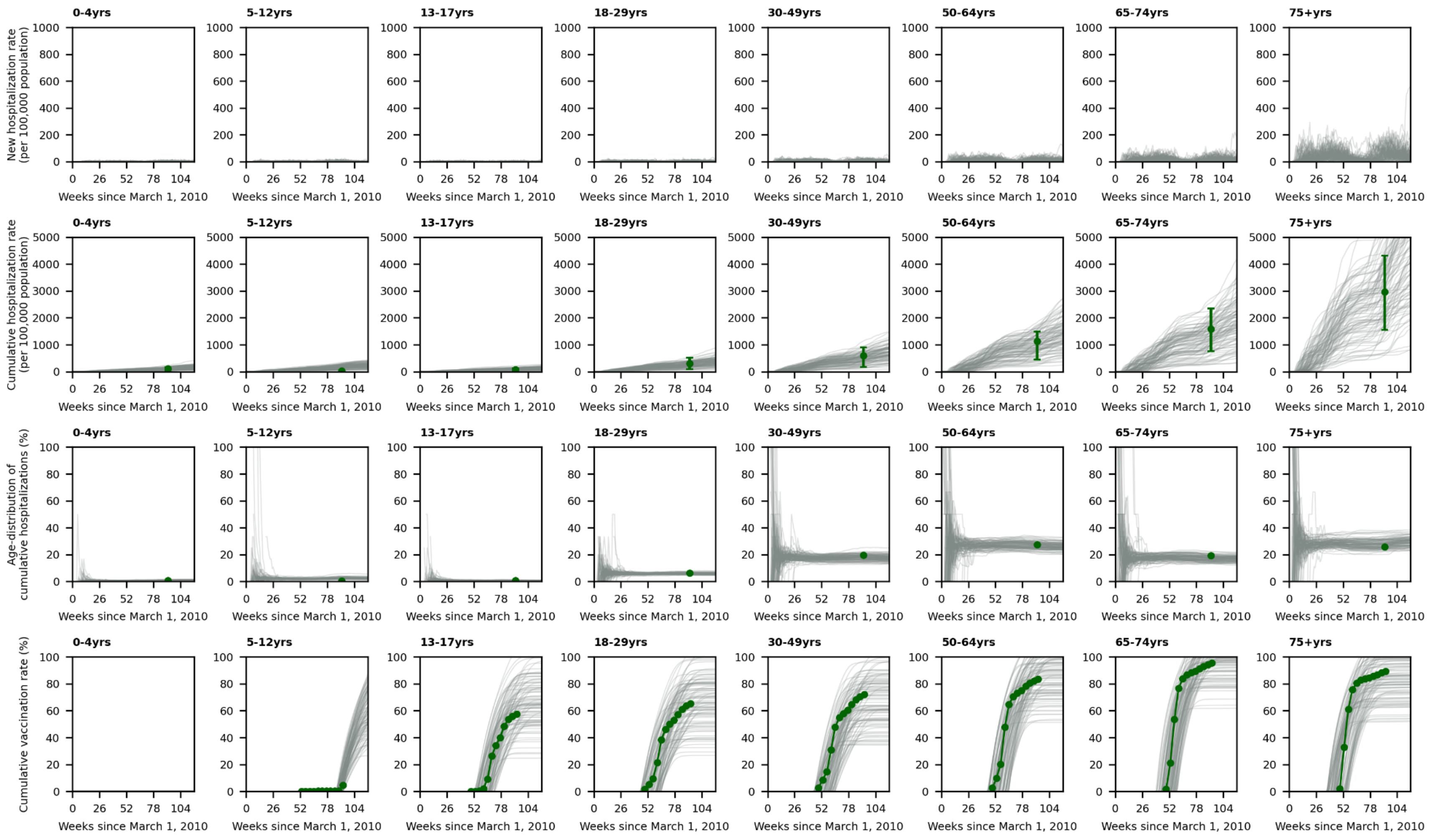

Fig. S5: Rates of weekly hospitalizations and cumulative hospitalizations, age-distribution of cumulative hospitalizations, and cumulative vaccination rates by age. The week 91 marks the beginning of winter 2022.

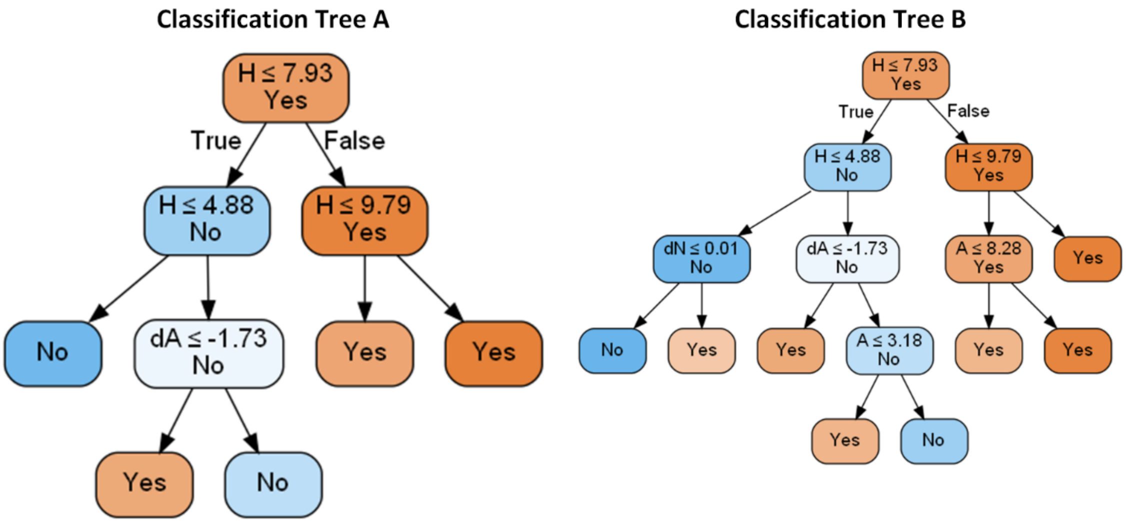

H: Current hospital occupancy due to COVID-19 (per 100,000 population).
A: Rate of weekly new COVID-19 hospital admission averaged over the past 2 weeks (per 100,000 population).
dH: Change in weekly new COVID-19 hospitalizations over the past 4 weeks (per 100,000 population).
dN: Change in weekly prevalence of novel variant among new infections over the past 4 weeks.

Fig. S6: Decision Trees A and B to predict whether the hospital occupancy due to COVID-19 would surpass the <u>threshold of 10</u> per 100,000 population within the next <u>8 weeks</u> during the winter and spring of 2022.

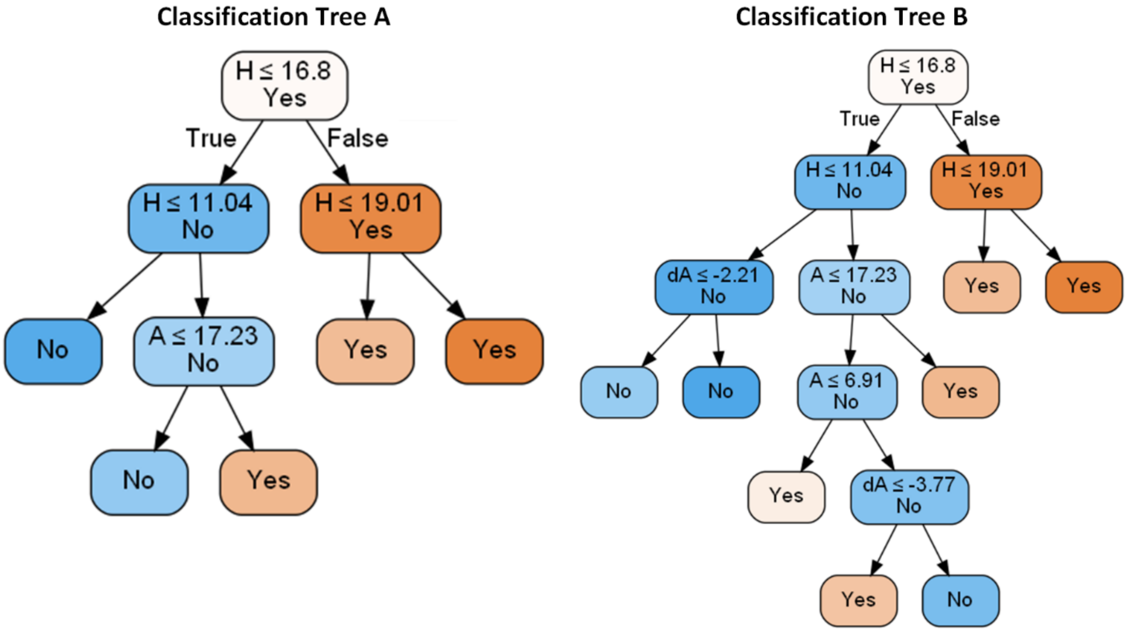

H: Current hospital occupancy due to COVID-19 (per 100,000 population).
A: Rate of weekly new COVID-19 hospital admission averaged over the past 2 weeks (per 100,000 population).
dH: Change in weekly new COVID-19 hospitalizations over the past 4 weeks (per 100,000 population).
dN: Change in weekly prevalence of novel variant among new infections over the past 4 weeks.

Fig. S7: Decision Trees A and B to predict whether the hospital occupancy due to COVID-19 would surpass the <u>threshold of 20</u> per 100,000 population within the next <u>8 weeks</u> during the winter and spring of 2022.

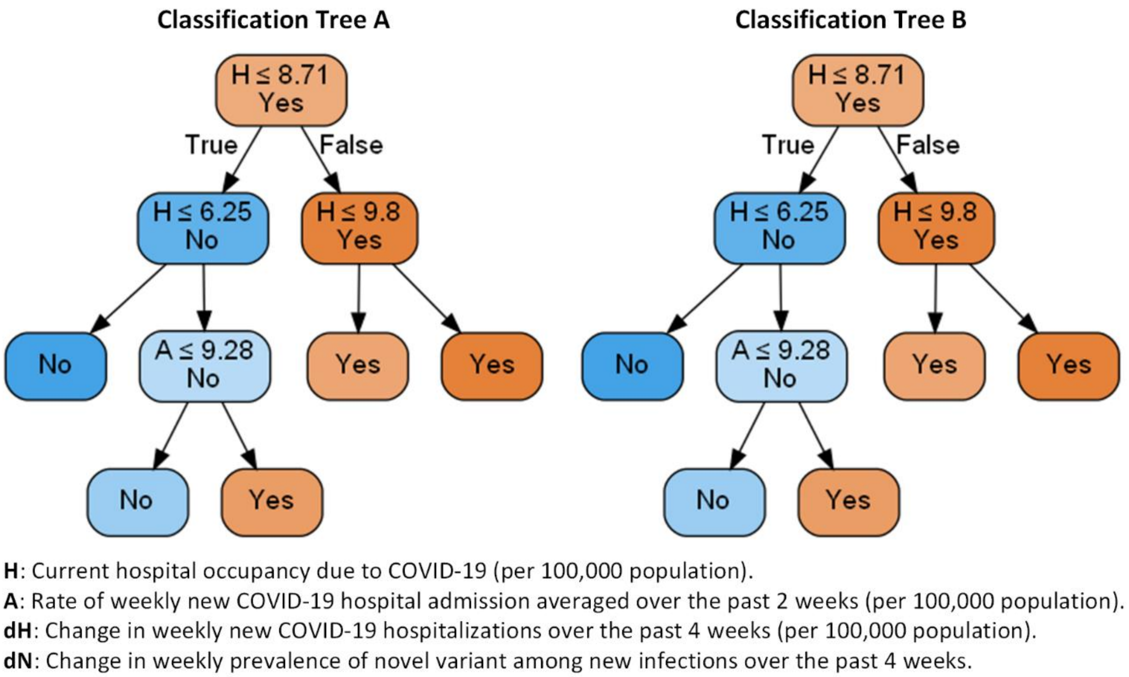

Fig. S8: Decision Trees A and B to predict whether the hospital occupancy due to COVID-19 would surpass the threshold of 10 per 100,000 population within the next 4 weeks during the winter and spring of 2022.

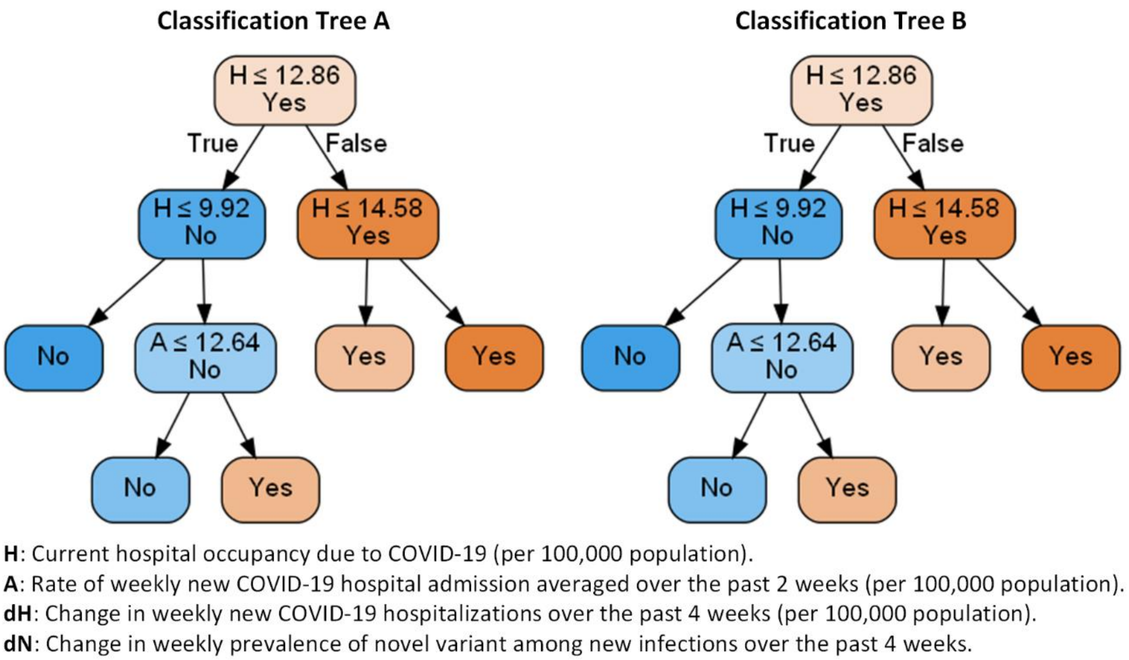

Fig. S9: Decision Trees A and B to predict whether the hospital occupancy due to COVID-19 would surpass the threshold of 15 per 100,000 population within the next 4 weeks during the winter and spring of 2022.

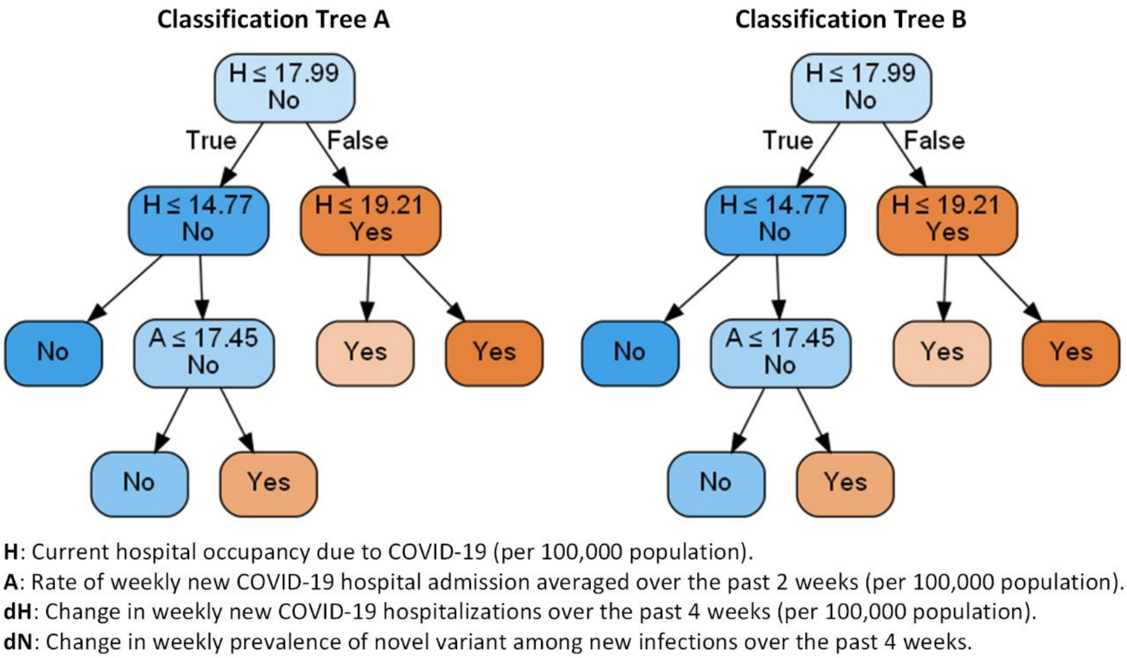

Fig. S10: Decision Trees A and B to predict whether the hospital occupancy due to COVID-19 would surpass the threshold of 20 per 100,000 population within the next 4 weeks during the winter and spring of 2022.

References

[1] statista. Resident population of the United States by sex and age as of July 1, 2019. 2021 June 25, 2021]Available from: https://www.statista.com/statistics/241488/population-of-the-us-by-sex-and-age/
[2] Prem K, Cook AR, Jit M. Projecting social contact matrices in 152 countries using contact surveys and demographic data. PLoS Comput Biol. 2017; 13(9):e1005697.
[3] Medlock J, Galvani AP. Optimizing influenza vaccine distribution. Science. 2009; 325(5948):1705-8.
[4] Kissler SM, Tedijanto C, Goldstein E, Grad YH, Lipsitch M. Projecting the transmission dynamics of SARS-CoV-2 through the postpandemic period. Science. 2020.
[5] Jarvis CI, Van Zandvoort K, Gimma A, Prem K, group CC-w, Klepac P, et al. Quantifying the impact of physical distance measures on the transmission of COVID-19 in the UK. BMC Med. 2020; 18(1):124.
[6] Saad-Roy CM, Wagner CE, Baker RE, Morris SE, Farrar J, Graham AL, et al. Immune life history, vaccination, and the dynamics of SARS-CoV-2 over the next 5 years. Science. 2020; 370(6518):811-8.
[7] Moghadas SM, Shoukat A, Fitzpatrick MC, Wells CR, Sah P, Pandey A, et al. Projecting hospital utilization during the COVID-19 outbreaks in the United States. P Natl Acad Sci USA. 2020; 117(16):9122-6.
[8] Pedregosa F, Varoquaux G, Gramfort A, Michel V, Thirion B, Grisel O, et al. Scikit-learn: Machine Learning in Python. J Mach Learn Res. 2011; 12:2825-30.
[9] Hastie T, Tibshirani R, Friedman JH. The elements of statistical learning : data mining, inference, and prediction. 2nd ed. New York: Springer 2009.
[10] Burkov A. The hundred-page machine learning book. Quebec City, Canada: Andriy Burkov 2019.
[11] Imai N, Cori A, Dorigatti I, Baguelin M, Donnelly CA, Riley S, et al. Report 3: Transmissibility of 2019-nCoV. Imperial College London 2020.
[12] Subramanian R, He Q, Pascual M. Quantifying asymptomatic infection and transmission of COVID-19 in New York City using observed cases, serology, and testing capacity. Proc Natl Acad Sci U S A. 2021; 118(9).
[13] Lauer SA, Grantz KH, Bi Q, Jones FK, Zheng Q, Meredith HR, et al. The Incubation Period of Coronavirus Disease 2019 (COVID-19) From Publicly Reported Confirmed Cases: Estimation and Application. Ann Intern Med. 2020.
[14] Chinazzi M, Davis JT, Ajelli M, Gioannini C, Litvinova M, Merler S, et al. The effect of travel restrictions on the spread of the 2019 novel coronavirus (COVID-19) outbreak. Science. 2020.
[15] Molteni E, Sudre CH, Canas LS, Bhopal SS, Hughes RC, Antonelli M, et al. Illness duration and symptom profile in symptomatic UK school-aged children tested for SARS-CoV-2. Lancet Child Adolesc Health. 2021.
[16] Guan WJ, Ni ZY, Hu Y, Liang WH, Ou CQ, He JX, et al. Clinical Characteristics of Coronavirus Disease 2019 in China. N Engl J Med. 2020.
[17] Dan JM, Mateus J, Kato Y, Hastie KM, Yu ED, Faliti CE, et al. Immunological memory to SARS-CoV-2 assessed for up to 8 months after infection. Science. 2021; 371(6529).
[18] Finelli L, Gupta V, Petigara T, Yu K, Bauer KA, Puzniak LA. Mortality Among US Patients Hospitalized With SARS-CoV-2 Infection in 2020. JAMA Netw Open. 2021; 4(4):e216556.
[19] Galloway SE, Paul P, MacCannell DR, Johansson MA, Brooks JT, MacNeil A, et al. Emergence of SARS-CoV-2 B.1.1.7 Lineage - United States, December 29, 2020-January 12, 2021. MMWR Morb Mortal Wkly Rep. 2021; 70(3):95-9.
[20] Borchering RK, Viboud C, Howerton E, Smith CP, Truelove S, Runge MC, et al. Modeling of Future COVID-19 Cases, Hospitalizations, and Deaths, by Vaccination Rates and Nonpharmaceutical Intervention Scenarios - United States, April-September 2021. MMWR Morb Mortal Wkly Rep. 2021; 70(19):719-24.
[21] Giles B, Meredith P, Robson S, Smith G, Chauhan A, Pacific, et al. The SARS-CoV-2 B.1.1.7 variant and increased clinical severity-the jury is out. Lancet Infect Dis. 2021.
[22] Twohig KA, Nyberg T, Zaidi A, Thelwall S, Sinnathamby MA, Aliabadi S, et al. Hospital admission and emergency care attendance risk for SARS-CoV-2 delta (B.1.617.2) compared with alpha (B.1.1.7) variants of concern: a cohort study. Lancet Infect Dis. 2021.

[23] Sheikh A, McMenamin J, Taylor B, Robertson C, Public Health S, the EIIC. SARS-CoV-2 Delta VOC in Scotland: demographics, risk of hospital admission, and vaccine effectiveness. Lancet. 2021; 397(10293):2461-2.

[24] Murray CJL, Piot P. The Potential Future of the COVID-19 Pandemic: Will SARS-CoV-2 Become a Recurrent Seasonal Infection? JAMA. 2021; 325(13):1249-50.

[25] Sandmann FG, Davies NG, Vassall A, Edmunds WJ, Jit M, Centre for the Mathematical Modelling of Infectious Diseases C-wg. The potential health and economic value of SARS-CoV-2 vaccination alongside physical distancing in the UK: a transmission model-based future scenario analysis and economic evaluation. Lancet Infect Dis. 2021; 21(7):962-74.

[26] Eyre DW, Taylor D, Purver M, Chapman D, Fowler T, Pouwels KB, et al. The impact of SARS-CoV-2 vaccination on Alpha & Delta variant transmission. medRxiv. 2021:2021.09.28.21264260.
